# Using proteomics to identify the mechanisms underlying the benefits of statins on ischemic heart disease

**DOI:** 10.1038/s44325-024-00018-6

**Published:** 2024-08-30

**Authors:** Jie V. Zhao, Junmeng Zhang

**Affiliations:** 1 School of Public Health, Li Ka Shing Faculty of Medicine, The University of Hong Kong, Hong Kong SAR, China; 2 State Key Laboratory of Pharmaceutical Biotechnology, The University of Hong Kong, Hong Kong SAR, China

**Keywords:** Cardiology, Cardiovascular diseases, Therapeutics

## Abstract

Ischemic heart disease (IHD) is the single leading cause of mortality globally. Statins are the mainstay for IHD treatment. However, the specific mechanisms underlying statins’ benefits on IHD have not been clarified. To examine the mechanisms through proteins, we used two-step Mendelian randomization (MR) approach. First, we examined the associations of genetically mimicked statins with 2923 proteins using genome-wide association of proteins from the UK Biobank Pharma Proteomics Project (UKB-PPP) to identify the proteins affected by statins, and replicated the findings using deCODE. Then we examined the associations of selected proteins with IHD risk using CARDIoGRAMplusC4D using MR, and replicated using FinnGen, and using another set of genetic instruments from deCODE. We selected proteins decreased or increased IHD risk and meanwhile increased or lowered by statins. We further examined the role of the selected protein(s) on common IHD comorbidities, including diabetes, chronic kidney disease (CKD), and kidney function (measured by estimated glomerular filtration rate (eGFR)). Nine proteins were affected by statins, including four proteins (PLA2G7, FGFBP1, ANGPTL1, and PTPRZ1) lowered by statins, and five proteins (EFNA4, COL6A3, ASGR1, PRSS8 and PCOLCE) increased by statins. Among these, PLA2G7 was related to higher risk of IHD after controlling for multiple testing. The associations were robust to different analytic methods and replication using another set of genetic instrument from deCODE, and using another GWAS of IHD from FinnGen. Genetically predicted PLA2G7 had null association with diabetes, CKD, and eGFR. We identified 9 proteins affected by statins, including 7 novel proteins which were not reported previously. PLA2G7 is on the pathway underlying statins’ benefits on IHD. The clarification of statins’ mechanisms had close relevance to precision medicine, and provided insights to the development of new treatment strategies.

## Introduction

Ischemic heart disease (IHD) is the single leading cause of mortality and poses a heavy burden on healthcare^1^. It accounts for ~16% of all deaths globally^2^, and ~ 19% of all deaths in Europe^3^. Lipid lowering therapy has been one of the main treatments for IHD. Since the discovery of statins in Japan in the early 1970s, statins have revolutionized the treatment of IHD^4^. Accumulating evidence from meta-analysis of randomized clinical trials has shown that statins can effectively lower cardiovascular mortality^5–7^, with superior efficacy in reducing cardiovascular mortality than other lipid-lowering drugs, such as ezetimibe^7,8^ and cholestyramine^8^. Notably, statins are also cost-effective for most people with even modestly elevated cholesterol or any coronary heart disease risk factors; the estimated cost is only $ 2 per month^9,10^. In all international guidelines, there is a consensus on the use of statins as the first-line treatment in primary and secondary prevention of atherosclerotic cardiovascular disease^9,11^.

Despite its wide use, the specific mechanism by which statins exert benefits on IHD have not been fully clarified. It is thought that the primary mechanism is via inhibiting hepatic cholesterol biosynthesis, upregulating the hepatic low-density lipoprotein (LDL) receptors and increasing the clearance of LDL-cholesterol^12^. However, the molecular pathways have not been clarified. Moreover, statins may act via pathways beyond LDL-cholesterol. In JUPITER, a primary prevention trial of rosuvastatin, the real benefits exceeded the expected benefit from LDL-cholesterol reduction on the Cholesterol Treatment Trialists’ (CTT) Collaboration regression line^8^, suggesting that statins may exert protective effects that are independent of LDL-cholesterol lowering^8^. Clarifying the mechanisms of statins is vital for understanding the pleiotropic effect of statins, and provides novel insights to new modifiable targets for drug development. Proteins are downstream factors of gene expression, play an important role in etiology of disease, including IHD^13^, and are often used as drug targets for treatment. However, the specific proteins mediating the cardiovascular benefits of statins have not been examined. Mechanistic, large-scale randomized controlled trials (RCTs) would be ideal to answer this question, but they are not available yet.

In this situation, Mendelian randomization (MR) studies, which can foreshadow the results of RCTs, could provide an alternative approach^14^. As genetic variants are randomly allocated at conception and unlikely affected by socioeconomic position or other confounders, the study design minimizes residual confounding^15^. In recent years, drug target MR has been widely applied to evaluate efficacy and safety of drugs targeting cardiovascular risk factors^16^, including statins^17–21^. However, no MR study has examined the molecular pathways underlying the protective effects of statins. The emerging large genome-wide association studies (GWAS) of proteins, such as GWAS of 2923 proteins in 54,219 participants in UK Biobank Pharma Proteomics Project (UKB-PPP)^22^, and GWAS of 4907 proteins in 35,559 Icelanders from deCODE^23^, provided a valuable opportunity to explore the mechanisms underlying the benefits of statins on IHD.

## Methods

### Study design

We used two-step MR to identify proteins on the pathway from statins to IHD.

First, we conducted MR analysis on the associations of genetically mimicked statins with 2923 proteins measured in UKB-PPP, to identify the proteins affected by statins. For replication, we examined the associations of genetically mimicked statins with selected proteins using another GWAS from deCODE^23^.

Second, we used MR to examine the effect of selected proteins on the risk of IHD using CARDIoGRAMplusC4D^24^. We also replicated the analysis using FinnGen. For replication, we also used another set of genetic instruments extracted from the GWAS from deCODE^23^, and examined the role of selected proteins in IHD.

Then, combining the two steps, we identified proteins that (1) lowered the risk of IHD and were increased by statins; or (2) increased the risk of IHD and were lowered by statins.

**Fig. 1:**
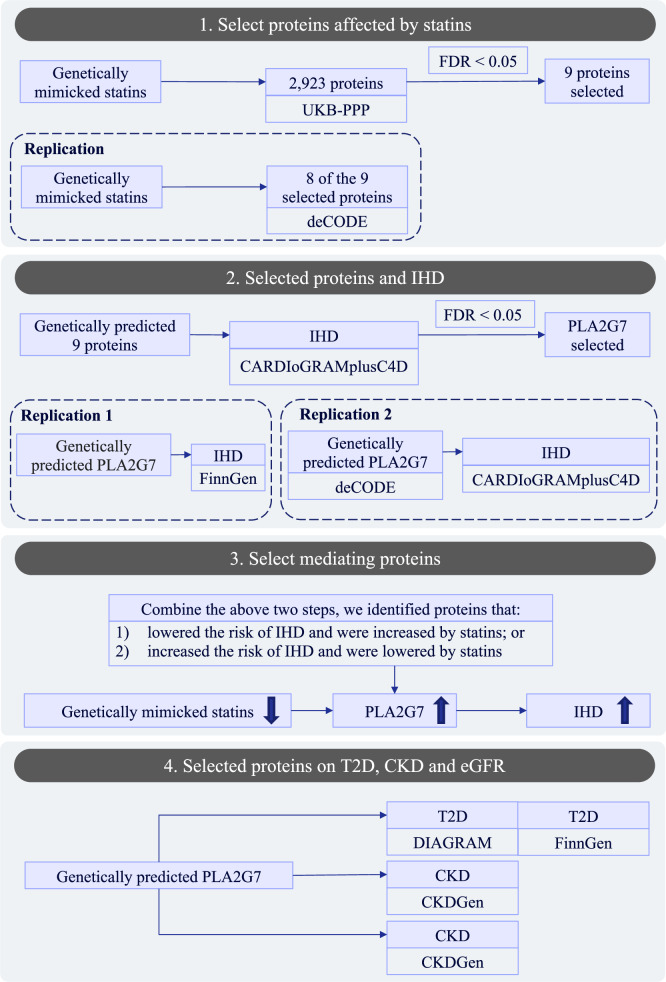
Flow chart of the two-step MR study design to identify proteins on the pathway from statins to IHD. UKB-PPP UK Biobank Pharma Proteomics Project, FDR False Discovery Rate, IHD Ischemic Heart Disease, CARDIoGRAMplusC4D “Coronary ARtery DIsease Genome-wide Replication and Meta-analysis (CARDIoGRAM) plus The Coronary Artery Disease (C4D) Genetics” consortium, T2D Type 2 Diabetes, CKD Chronic Kidney Disease, eGFR Estimated Glomerular Filtration Rate, DIAGRAM Diabetes Genetics Replication and Meta-analysis consortium, CKDGen Chronic Kidney Disease Genetics Consortium

To test whether the identified target(s) have pleiotropic effects on common comorbidities of IHD, including diabetes and chronic kidney disease (CKD), we also examined the effects of the target(s) on diabetes, CKD and kidney function (indicated by eGFR). The flow chart was shown in Fig. 1. The data sources we used were shown in Supplemental Table 1.

In the following sections we provided details of each step.

### Associations of genetic proxies for statins with proteins

As previously^19,20^, genetic proxies for statins were genetic variants in the drug target gene, *HMGCR*, and also associated with lower LDL-cholesterol. Specifically, the previous studies selected SNPs using the following criteria: (1) in *HMGCR* or within 100 kb on either side of *HMGCR*, and (2) were associated with LDL-cholesterol at genome-wide significance. For the second criteria, the associations with LDL-cholesterol were checked in GWAS of LDL-cholesterol in up to 173,082 participants of European ancestry from the Global Lipids Genetic Consortium (GLGC)^25^. To ensure the genetic variants are independent, as previously^20^, we selected SNPs not in linkage disequilibrium (LD) (*r*^2^ < 0.05), and used the lead SNP (rs12916) as the genetic instrument. To check for the potential pleiotropy, we looked into GWAS Catalog (https://www.ebi.ac.uk/gwas/variants/rs12916), which includes comprehensive genotype-phenotype associations. As expected, rs12916 was associated with lipid traits but not with potential confounders.

Then we obtained the associations of the genetic instrument with 2923 proteins measured in UKB-PPP. UK Biobank is a large, population-based cohort in UK, with around 500,000 participants aged 40–69 years, recruited between 2006 and 2010^26^. UKB-PPP is a precompetitive consortium of 13 biopharmaceutical companies which funded the generation of multiplex proteomic data in 54,219 UKB participants. The details of UKB-PPP were shown in the previous publication^22^. A total of 2923 unique proteins across eight protein panels (cardiometabolic, cardiometabolic II, inflammation, inflammation II, neurology, neurology II, oncology and oncology II) in the antibody-based Olink Explore 3072 platform were measured, with quality control^22^. One protein (GLIPR1) failed quality control and was excluded^22^, so 2922 proteins were included in the analysis.

In the first-step MR, we obtain Wald ratio, i.e., the genetic association with each protein divided by the association with the reduction in LDL-cholesterol. As statins are lipid-lowering drugs, the reduction in LDL-cholesterol is used as a scale to measure the effects of statins. As we did multiple testing, to control the ratio of false positive results to true positive results within a certain range, we selected proteins with false discovery rate (FDR) < 0.05. For replication, we used another GWAS in deCODE^23^, a GWAS of 4907 proteins in 35,559 Icelanders, to examine the role of statins on selected protein(s).

### The effect of selected proteins on the risk of IHD

The genetic variants for each selected protein were obtained from the published study in *Nature*^22^, which provided SNPs strongly associated with the proteins (*p* < 1.7 × 10^−11^) and not in linkage disequilibrium (*r*^*2*^ < 0.01). Then we obtained the genetic associations with IHD, using a GWAS meta-analysis from Coronary ARtery DIsease Genome wide Replication and Meta-analysis (CARDIoGRAM) plus The Coronary Artery Disease (C4D) Genetics (CARDIoGRAMplusC4D) including UK Biobank SOFT CAD GWAS (10,801cases and 137,371 controls), CARDIoGRAMplusC4D 1000 Genomes-based GWAS (60,801 CAD cases and 123,504 controls) and the Myocardial Infarction Genetics and CARDIoGRAM Exome (42,335 cases and 78,240 controls)^24^. We also replicated the analysis using FinnGen (30,952 cases and 187,840 controls), and combined the MR estimates from the two data sources.

In the second-step MR, we obtained Wald ratio estimates for each SNP, using genetic associations with IHD divided by genetic associations with each protein, and meta-analysed these estimates using inverse variance weighting (IVW). Multiplicative random effects model was used when three or more genetic variants were used as instruments, and fixed effects model was used when less than three genetic variants were used as instruments. To control for multiple testing, we also selected proteins associated with IHD with FDR < 0.05.

### Sensitivity analyses

For replication, we used another set of genetic instruments for the selected proteins, where we selected SNPs with genome-wide significance, and not in linkage disequilibrium (*r*^*2*^ < 0.001) from the GWAS of proteins in deCODE^23^. In sensitivity analysis, to visualize the potential outliers, we used leave-one-out analysis and scatter plots. For proteins with three or more genetic variants as instruments, we also used different MR methods under different assumptions from IVW, including weighted median, weighted mode and Mendelian Randomization Pleiotropy RESidual Sum and Outlier (MR-PRESSO). The weighted median method can provide consistent estimates even when up to 50% of the information comes from invalid genetic variants^27^. The weighted mode is based on the assumption that a plurality of genetic variants are valid instruments, i.e., no larger subset of invalid instruments estimating the same causal parameter than the subset of valid instruments exists^28^. MR-PRESSO was able to identify the outliers among the genetic variant(s) which differentially drive the associations^29^, and provide corrected estimates removing the outliers. We also conducted reverse MR analyses for the proteins associated with IHD or having suggestive associations with IHD, to examine whether genetically predicted IHD was associated with the proteins. To build the genetic instrument for IHD, we selected SNPs with genome-wide significance and not in linkage disequilibrium (*r*^2^ < 0.001) in the GWAS of IHD from CARDIoGRAMplusC4D^24^. In the reverse MR, similarly we used IVW, weighted median, weighted mode and MR-PRESSO. We also used Steiger test which can infer the causal direction, by calculating and comparing the variance explained by the genetic instrument in exposure (here IHD) and in outcome (here the proteins)^30^.

To exclude the possible confounding by genotype, we conducted colocalization analysis for the effect of statins on the selected protein. In contrast to MR analysis which only used genetic variants strongly associated with the exposure, when conducting the colocalization analysis, we used all the genetic variants within +/-200 kb of the *HMGCR* gene, and then obtained their associations with LDL-cholesterol and with the selected protein. Then we conducted the colocalization analysis using the “coloc” package^31^ in R. Colocalization tested four hypotheses: H_0_: no association with either trait; H_1_: association with only trait 1; H_2_: association with only trait 2; H_3_: two independent SNPs associated with both traits; H_4_: the same SNP associated with both traits. A high PP_H3_ suggests confounding^32^.

### Selection of mediating proteins based on the prior steps and replication

Based on the prior two-step MR, we identified proteins potentially mediating the role of statins in IHD, i.e., proteins (1) affected by statins and also affect the risk of IHD and (2) the directions of associations with statins and IHD were consistent with the beneficial association of statins with IHD. The proportion of mediation was calculated using indirect effect divided by total effect. The total effect was the effect of statins on IHD. The indirect effect was calculated using the estimates from the two-step MR^33^, i.e., the association of statins with the selected protein multiplied by the association of selected protein with IHD.

### The effects of the identified protein(s) on type 2 diabetes, CKD and eGFR

For the identified mediating proteins, to examine their pleiotropic effects, we also examined their associations with diabetes, CKD and eGFR. Genetic associations with diabetes were obtained from DIAGRAM (74,124 cases and 824,006 controls)^34^ and FinnGen (32,469 cases and 183,185 controls) (Supplemental Table 1). Similarly, the MR estimates for diabetes from the two GWAS were meta-analyzed. Genetic associations with CKD and eGFR (calculated based on creatinine) were obtained from CKDGen Consortium. The GWAS meta-analysis of CKD included 41,395 cases and 439,303 controls in 23 studies; all are of European ancestry^35^. The GWAS meta-analysis of eGFR included up to 567,460 participants in 42 studies^35^. Similar to prior analyses, we used IVW in the main analyses, supplemented by sensitivity analyses using weighted median, weighted mode and MR-PRESSO. To be comprehensive, we also examine the pleiotropic effects of proteins that are affected by statins and also affect the risk of IHD, but the directions of associations with statins and IHD were inconsistent with the beneficial association of statins with IHD.

All statistical analyses were conducted using the “TwoSampleMR”^36^, “MendelianRandomization”^37^, “MRPRESSO”^38^ and “coloc”^31^ packages in R (version 4.0.1, R Foundation for Statistical Computing, Vienna, Austria).

## Results

### Associations of genetic proxies for statins with proteins

**Fig. 2:**
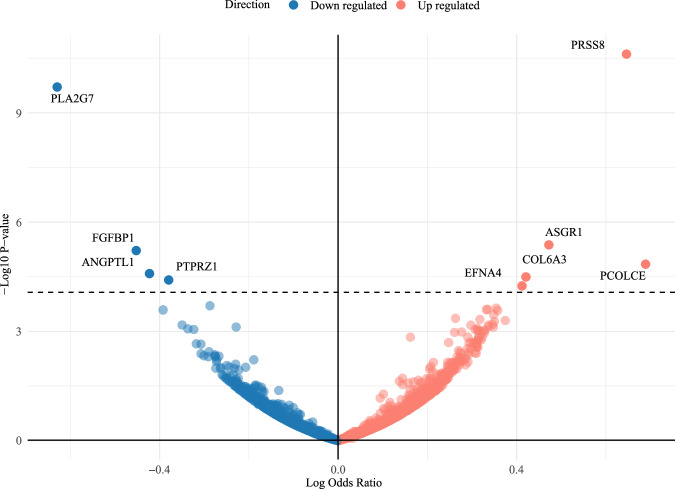
Volcano plot of the MR analysis examining the associations of genetically mimicked statins with 2923 proteins to identify those affected by statins. The *y*-axis is the negative log10 (false discovery rate-adjusted *P* value), the *x*-axis is the Mendelian randomization estimates of the associations of statins with 2923 proteins.

In the analysis on genetically mimicked statins with proteins (shown in Fig. 2), after FDR, 9 proteins were identified (Table 1), including 4 proteins (PLA2G7, FGFBP1, ANGPTL1, and PTPRZ1) lowered by statins, and 5 proteins (EFNA4, COL6A3, ASGR1, PRSS8 and PCOLCE) increased by statins. In the replication analysis, one protein (PTPRZ1) was not able to be replicated due to lack of data. For the 8 proteins with replication available, we replicated the associations of statins with 4 proteins, including PLA2G7, FGFBP1, PRSS8 and PCOLCE (Table 2).

**Table 1:** The associations of genetically mimicked statins with nine proteins selected from proteome-wide Mendelian randomization analysis

Protein	beta (95% CI)	*p*-value
ANGPTL1	−0.42 (−0.62, −0.23)	2.61 × 10^−5^
ASGR1	0.47 (0.27, 0.67)	4.24 × 10^−6^
COL6A3	0.42 (0.22, 0.62)	3.23 × 10^−5^
EFNA4	0.41 (0.21, 0.61)	6.16 × 10^−5^
FGFBP1	−0.45 (−0.65, −0.26)	6.08 × 10^−6^
PCOLCE	0.69 (0.38, 1.00)	1.44 × 10^−5^
PLA2G7	-0.63 (−0.82, −0.44)	1.92 × 10^−10^
PRSS8	0.65 (0.46, 0.84)	2.39 × 10^−11^
PTPRZ1	-0.38 (−0.56, −0.20)	3.92 × 10^−5^

*CI* confidence interval.

**Table 2:** Replication of the associations for 8 proteins changed by statins in deCODE

Protein	beta (95%CI)	*p*-value
ANGPTL1	0.01 (-0.20, 0.21)	0.96
ASGR1	0.03 (-0.19, 0.26)	0.77
COL6A3	-0.07 (-0.28, 0.14)	0.52
EFNA4	0.10 (-0.12, 0.32)	0.37
FGFBP1	-0.27 (-0.50, -0.04)	0.02
PCOLCE	0.47 (0.25, 0.69)	3.70 × 10^−5^
PLA2G7	-0.47 (-0.71, -0.23)	9.42 × 10^−5^
PRSS8	0.44 (0.21, 0.66)	1.29 × 10^−4^

### The effect of selected proteins on the risk of IHD

**Fig. 3:**
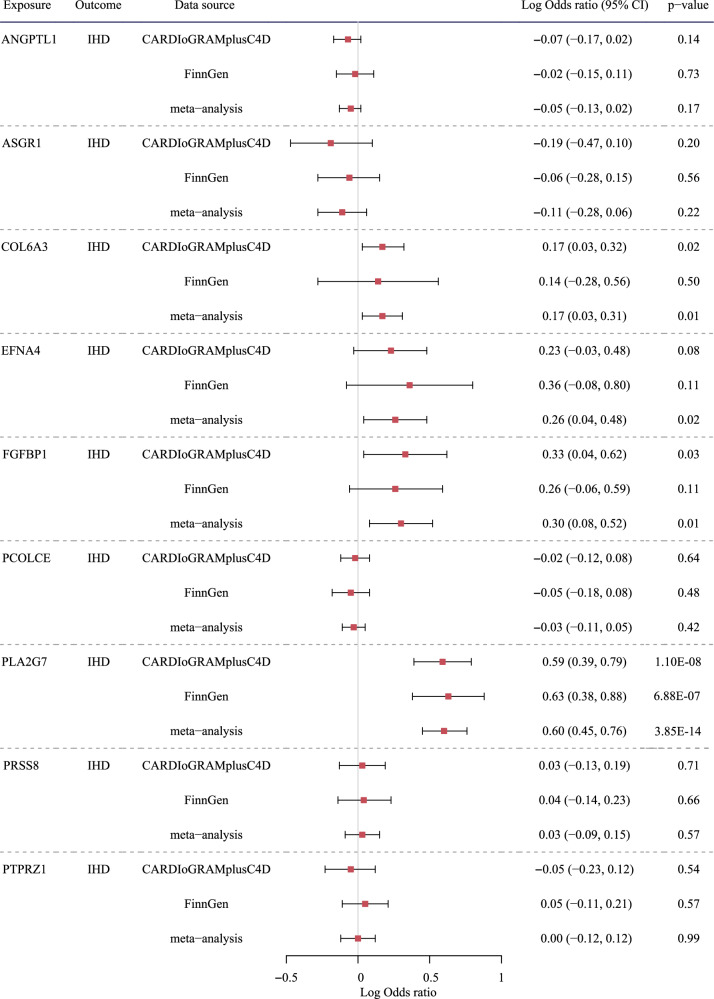
The association of 9 proteins with IHD in CARDIoGRAMplusC4D and FinnGen using IVW. IHD ischemic heart disease, CARDIoGRAMplusC4D Coronary ARtery DIsease Genome-wide Replication and Meta-analysis (CARDIoGRAM) plus The Coronary Artery Disease (C4D) Genetics consortium.

Then we examined the effect of the 9 proteins identified in UKB-PPP on IHD. All the SNPs for these selected proteins have F-statistics above 10 (genetic instruments shown in Supplemental Table 2). Of these 9 proteins, after multiple testing correction, only one protein, PLA2G7, remained to be related to IHD in CARDIoGRAMplusC4D. The associations were robust to different analytic methods (Supplemental Table 3). The positive association with IHD was replicated in FinnGen and in the meta-analysis of both GWAS (Fig. 3). In the replication using another set of genetic instruments derived from deCODE (Supplemental Table 4), the positive association of PLA2G7 with IHD was also replicated (Supplemental Table 5). Some outliers were detected in MR-PRESSO (Supplemental Table 6), scatter plots (Supplemental Figs. 1–9) and leave-one-out plots (Supplemental Figs. 10–18), but MR-PRESSO estimates removing the outliers showed consistent estimates as in the main analyses (Supplemental Table 3). In colocalization analysis, the small PP_H3_ (0.01) did not suggest the effect of statins on PLA2G7 is due to confounding by genotype. The proportion mediated by PLA2G7 was 77%, calculated based on the indirect effect via PLA2G7 (log odds ratio (OR) -0.38) and total effect (log OR -0.49, shown in Supplemental Table 7). Using a different dataset, i.e., for the effect of statins on PLA2G7, we used another dataset of PLA2G7 in deCODE, which does not overlap with UK Biobank, and for the effect of PLA2G7 on IHD, we used another dataset of IHD in FinnGen, which does not overlap with CARDIoGRAMplusC4D, we still found statins lowered PLA2G7 (Table 2), and PLA2G7 increased the risk of IHD (Fig. 3), the indirect effect via PLA2G7 is log OR -0.30, and the mediation proportion is 60%. Three proteins (FGFBP1, COL6A3 and EFNA4) had suggestive associations with IHD (Fig. 3), i.e., the associations with *p*-value < 0.05 but did not remain after multiple testing correction. PLA2G7 and FGFBP1 were related to higher risk of IHD and decreased by statins, consistent with statins lowering the risk of IHD. COL6A3 and EFNA4 were related to higher risk of IHD, and were increased by statins (Table 1), which does not fit with the inverse association of statins with IHD. In the reverse MR analysis, based on 49 SNPs for IHD, genetically predicted IHD was not related to the four proteins associated with IHD or had suggestive associations with IHD using IVW after multiple testing correction (Supplemental Table 8). Genetically predicted IHD was related to lower FGFBP1 and higher PLA2G7 in sensitivity analyses (Supplemental Table 8), but the Steiger test did not support the direction from IHD to proteins.

### The effects of the identified protein(s) on type 2 diabetes, CKD and eGFR

**Fig. 4:**
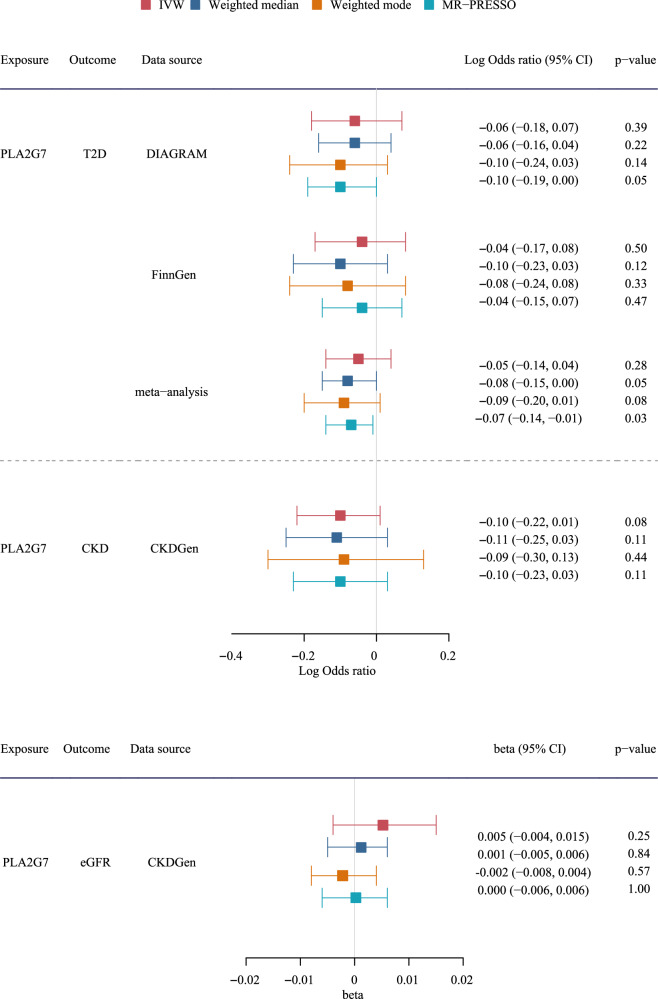
The association of PLA2G7 with diabetes, CKD and kidney function (eGFR) using various methods. T2D Type 2 Diabetes, CKD Chronic Kidney Disease, eGFR Estimated Glomerular Filtration Rate, DIAGRAM Diabetes Genetics Replication and Meta-analysis consortium, CKDGen Chronic Kidney Disease Genetics Consortium.

When assessing the role of PLA2G7 in diabetes, CKD and kidney function (eGFR), we found genetically predicted PLA2G7 was not related to diabetes, CKD and eGFR using IVW, and these findings were robust across different analytic methods (Fig. 4). Scatter plots and leave-one-out plots did not indicate outliers (Supplemental Figs. 19–21). The power calculation results were shown in Supplemental Table 9; at the current sample size, we can detect an effect size of OR 1.07–1.12 for the role of PLA2G7 in diabetes and CKD, and an effect size of 0.03 for eGFR. We also assessed the role of COL6A3 and EFNA4 in diabetes, CKD and eGFR (Supplemental Table 10). Genetically predicted COL6A3 was associated with lower risk of diabetes using IVW and weighted median, EFNA4 was related to higher risk of CKD and lower eGFR using weighted median, but the findings were not shown in other methods (Supplemental Table 10).

## Discussion

Our findings added to the limited evidence on the biological mechanism underlying statins’ cardiovascular benefits, by showing that PLA2G7 possibly mediated the effect of statins on IHD. Using proteomics in the MR analysis, we found that statins lowered PLA2G7, and genetically predicted PLA2G7 was related to higher risk of IHD. The associations were replicated using another GWAS of proteomics, and using a different set of genetic instrument for PLA2G7.

Our finding of statins lowering PLA2G7 were consistent with a previous clinical trial showing PLA2G7 decreased after taking statins^39^. Our finding of PLA2G7 increasing the risk of IHD is also consistent with a previous MR study^40^ showing lipoprotein-associated phospholipase A2 (Lp-PLA2) activity, coded by *PLA2G7* gene, was related to higher risk of IHD, which used genetic instruments different from our study. There is also genetic evidence suggests that Lp-PLA2-lowering alleles were not related to the risk of IHD^41^, however, this study was only conducted for each allele, rather than an MR study taking advantage of all genetic variants for PLA2G7. In addition, two large RCTs of darapladib, an Lp-PLA2 inhibitor, showed that in patients with coronary heart disease, darapladib did not reduce the risk of major coronary events^42,43^. It is possible that darapladib had unidentified off-target effects which may account for the absence of efficacy in the trials^42^. It is also possible that the clinical trials had a relatively short follow-up (mean follow-up time no more than 3.5 years in the two trials^42,43^), whilst MR examines the lifelong effect of PLA2G7. PLA2G7 hydrolyzes and inactivates platelet-activating factor (PAF), a potent pro-inflammatory signaling lipid, and hydrolyzes oxidatively truncated phospholipids carrying an aldehyde group at omega position, preventing their accumulation in LDL particles^44^. Recently, PLA2G7 has been recognized as a new player in shaping energy metabolism and lifespan^45^. Energy metabolism is related to multiple diseases, including cardiovascular disease, which may also explain the harmful effect of PLA2G7 on IHD. Notably, PLA2G7 was not related to diabetes, in contrast to statins increasing the risk of diabetes^46^. This suggests that the pathways underlying statins’ cardiovascular benefits are different from those underlying statins’ effect on diabetes. On the other hand, it suggests that drugs targeting PLA2G7 may lower the risk of IHD, without increasing the risk of diabetes, with relevance to new drug development.

In addition to PLA2G7, we also identified several proteins affected by statins, and three (FGFBP1, PRSS8 and PCOLCE) were replicated using another GWAS of proteins. Although they may not be the mediators for the cardiovascular benefit of statins, the identification of these targets may improve the understanding of statins’ effects, with relevance to the exploration of drug off-target effects. The increase in PCOLCE in our study was corroborated by the clinical trial showing PCOLCE was increased by taking statins^39^. To our knowledge, the effect of statins on FGFBP1 and PRSS8 has not been reported previously. FGFBP1 showed suggestive association with IHD, and also lowered by statins. It may also be involved in the pathway underlying statins’ cardiovascular benefits. Although there is no direct evidence on how statins affect FGFBP1, some studies suggest that statins may affect the expression of fibroblast growth factors (FGFs) and their binding proteins^47,48^. For example, one study showed that statins induced phosphorylation of the FGF receptor (FGFR), thereby exert proangiogenic effects, and the effect of statins was abolished by FGFR inhibitor^47^. Another study found that statins can decrease the expression of FGF-2^48^, which FGFBP1 binds to. It is possible that the effects of statins on FGFR and FGF-2 expression may indirectly affect the activity of FGFBP1, as FGFBP1 bind to them to exert an effect^49^. More studies are needed to clarify the mechanism by which statins affected FGFBP1.

Our study is the first study to use proteomics to examine the underlying pathways of statins’ benefits on IHD. MR can minimize confounding, thereby providing a more robust estimate than conventional observational study. MR also provides a cost-effective solution in the absence of large mechanistic RCTs. Despite the novelty, we acknowledge we had several limitations. First, MR is based on three core assumptions, i.e., the relevance, independence, and exclusion-restriction assumption^50^. To satisfy these assumptions, we used genetic variants strongly associated with these proteins. Considering the potential pleiotropy, we not only used multiple analytic methods robust to pleiotropy but also conducted replication using different datasets. As the confounding structure is expected to be different in different datasets, the replication in a different dataset can add confidence to the validity of the results. The findings robust to different analytic methods and replication analyses are less like to be due to pleiotropy. Second, population stratification might affect MR estimates. However, the GWAS data used in this study were derived from people largely of European ancestry. Meanwhile, as the study was based on people of European ancestry, the findings may not be generalizable to other ancestries. Third, it is possible that a small proportion of samples overlap in the GWAS of proteins and GWAS of outcomes (such as GWAS from CARDIoGRAMplusC4D, DIAGRAM, CKD and eGFR), which both included UK Biobank. The sample overlapping may bias the estimates in the MR analyses on the role of proteins in the outcomes^51^, however, a simulation study has shown that it is not a major concern if MR is conducted in large cohorts, such as UK Biobank^52^. There is no overlapping in the GWAS of exposure (i.e., the GWAS used for building genetic instrument for statins, from GLGC) and GWAS of the outcomes. Fourth, we conducted the analyses on all the proteins available in the proteomics panel, however, we did not identify apolipoprotein B (ApoB) to be affected by statins using the proteomics data in the UK Biobank (*n* = ~35,000). Despite, when using a much larger dataset, the UK Biobank metabolomics dataset (*n* = ~388,022), as expected, we found genetically mimicked statins lowered ApoB (beta -0.75, 95% CI -0.80 – -0.69, *p*-value = 2.8 × 10^−146^), which added validity to the genetic instrument for statins. As such, the lack of statistically significant association of the genetic instrument for statins with ApoB in the proteomics dataset may not be due to pleiotropy, but be due to the relatively small sample size of the proteomics dataset in UK Biobank. Fifth, we only identified one protein, PLA2G7, as a potential mediator after multiple testing correction, so we cannot use other related proteins to validate the result. However, the finding is supported by both biological plausibility and statical analysis (sensitivity analysis and replication analysis). From the perspective of biology, PLA2G7 is an enzyme involved in the metabolism of lipoprotein, including LDL. From the perspective of statistical analysis, to validate this finding, we have used several methods, including multiple analytic methods, replication using a different set of genetic instruments for PLA2G7 and replication using a different GWAS of IHD. The consistency from sensitivity analysis and replication analysis provided confidence to the validity of the finding. Sixth, the proportion of mediation via PLA2G7 varied when using different datasets in the analysis, which needs to be interpreted with caution. However, the mediation via PLA2G7 was replicated using studies with no overlapping. Finally, the selection of proteins was based on the proteins assayed in the UK Biobank. We cannot exclude the possibility that other proteins not assayed were involved in the mechanisms underlying statins’ cardiovascular effects.

From the perspective of public health and clinical perspective, our study suggests that PLA2G7 possibly mediated the role of statins in IHD. As such, medications or dietary factors affecting PLA2G7 are expected to exert cardiovascular benefits. We also identified several proteins affected by statins, including four proteins replicated in a different GWAS. FGFBP1 may also be relevant to statins’ biological pathways, but the effect of FGFBP1 on IHD remains to be replicated in large GWAS of IHD.

To sum up, we identified 9 proteins affected by statins, including 7 novel proteins which were not reported previously. We also found one protein (PLA2G7) on the pathway from statins to IHD. The clarification of the pathways underlying statins’ cardiovascular benefits had close relevance to precision medicine, and provided insights to the development of new treatment strategies.

## Supplementary information

Supplementary Information

## Data Availability

Data is provided within the manuscript or supplementary information files.
