# Supplementary material for: Using proteomics to identify the mechanisms underlying the benefits of statins on ischemic heart disease
Source: NPJ Cardiovasc Health. 2024 Aug 30;1:15. doi: 10.1038/s44325-024-00018-6 (PMC12912335; doi:10.1038/s44325-024-00018-6)
Supplement: Supplementary file 1 — Supplementary Information [file 44325_2024_18_MOESM1_ESM.pdf]

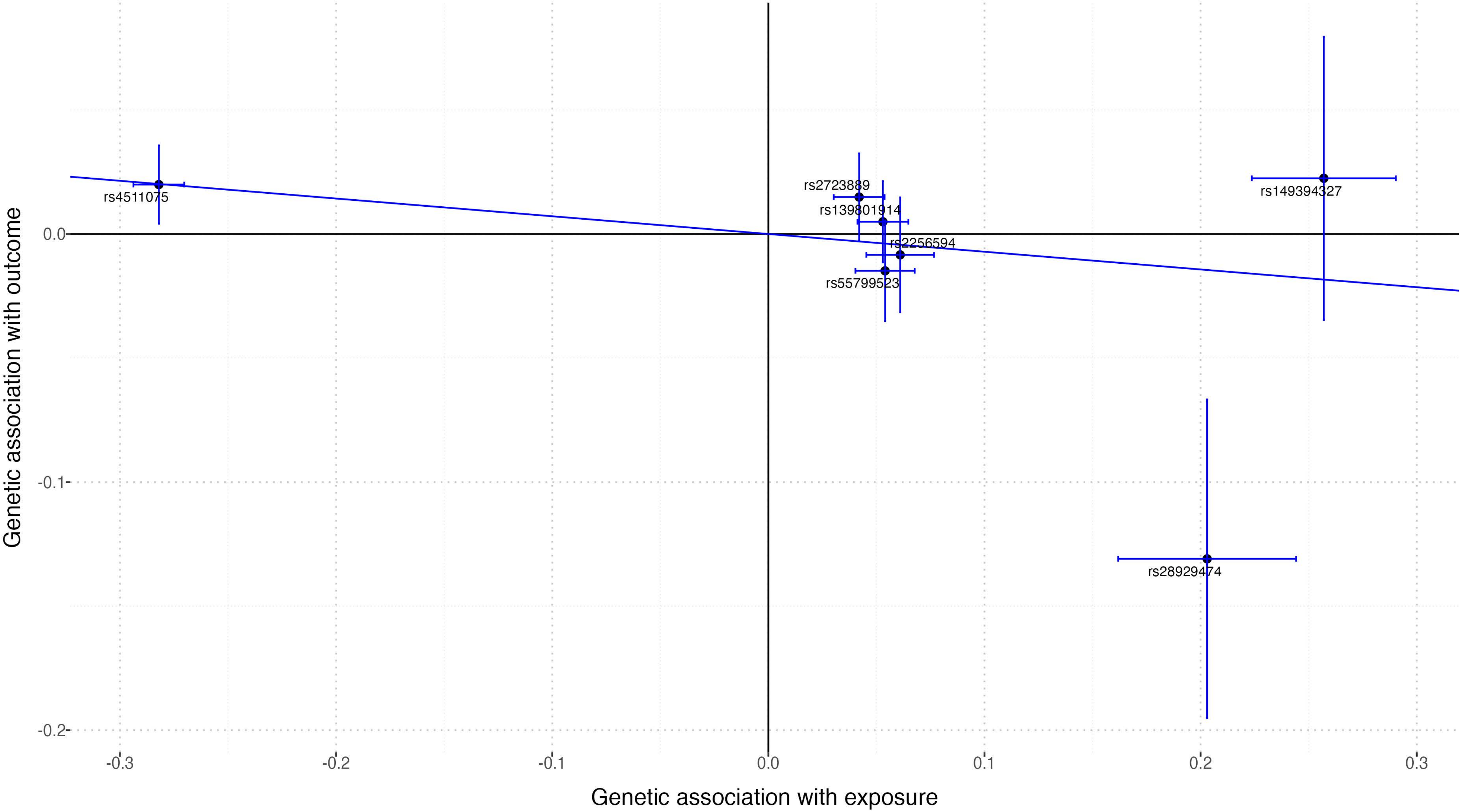

Supplemental Figure 1. Scatter plot showing the association of each SNP with ANGPTL1 and with IHD in CARDIoGRAMplusC4D.

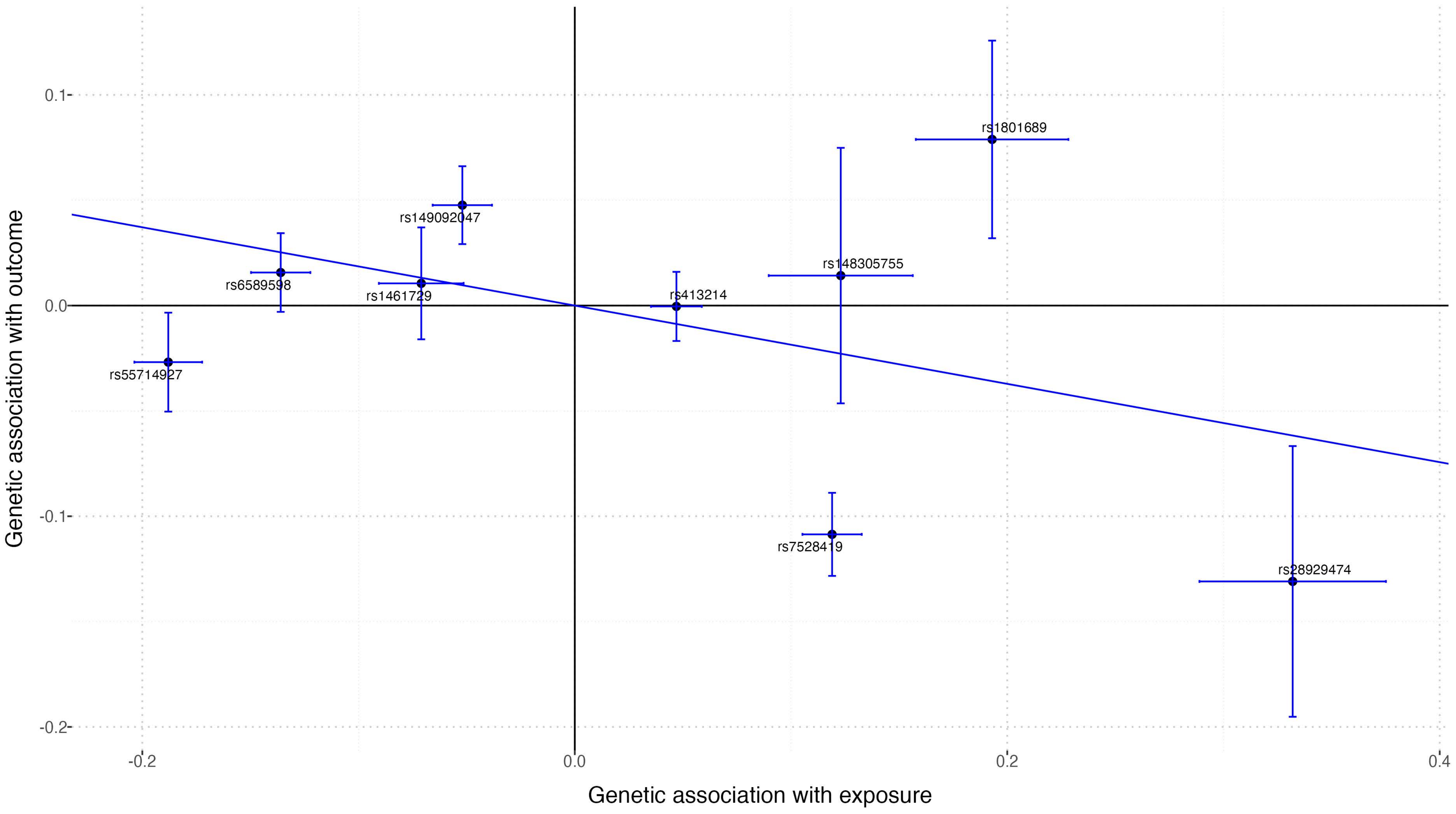

Supplemental Figure 2. Scatter plot showing the association of each SNP with ASGR1 and with IHD in CARDIoGRAMplusC4D.

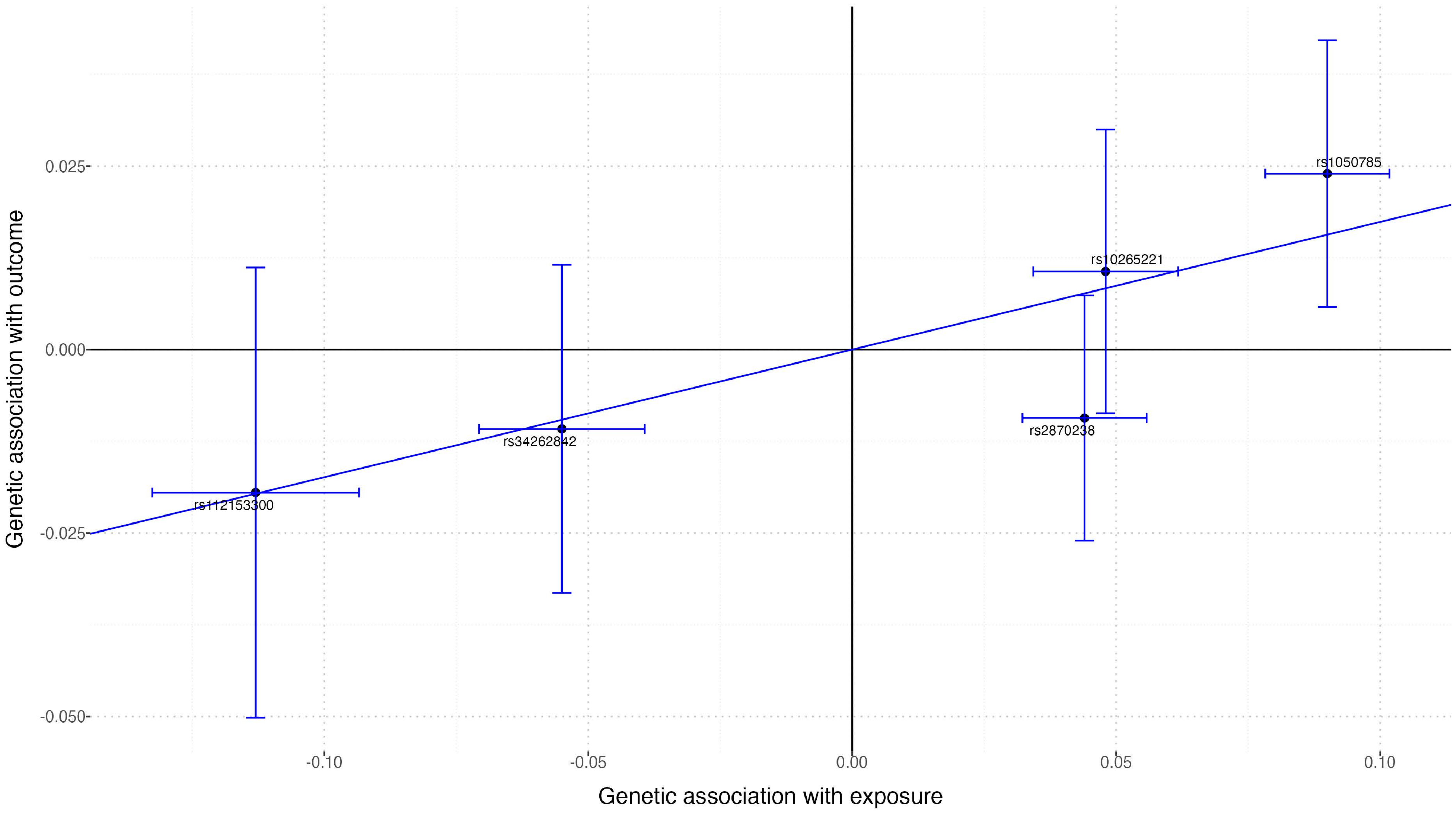

Supplemental Figure 3. Scatter plot showing the association of each SNP with COL6A3 and with IHD in CARDIoGRAMplusC4D.

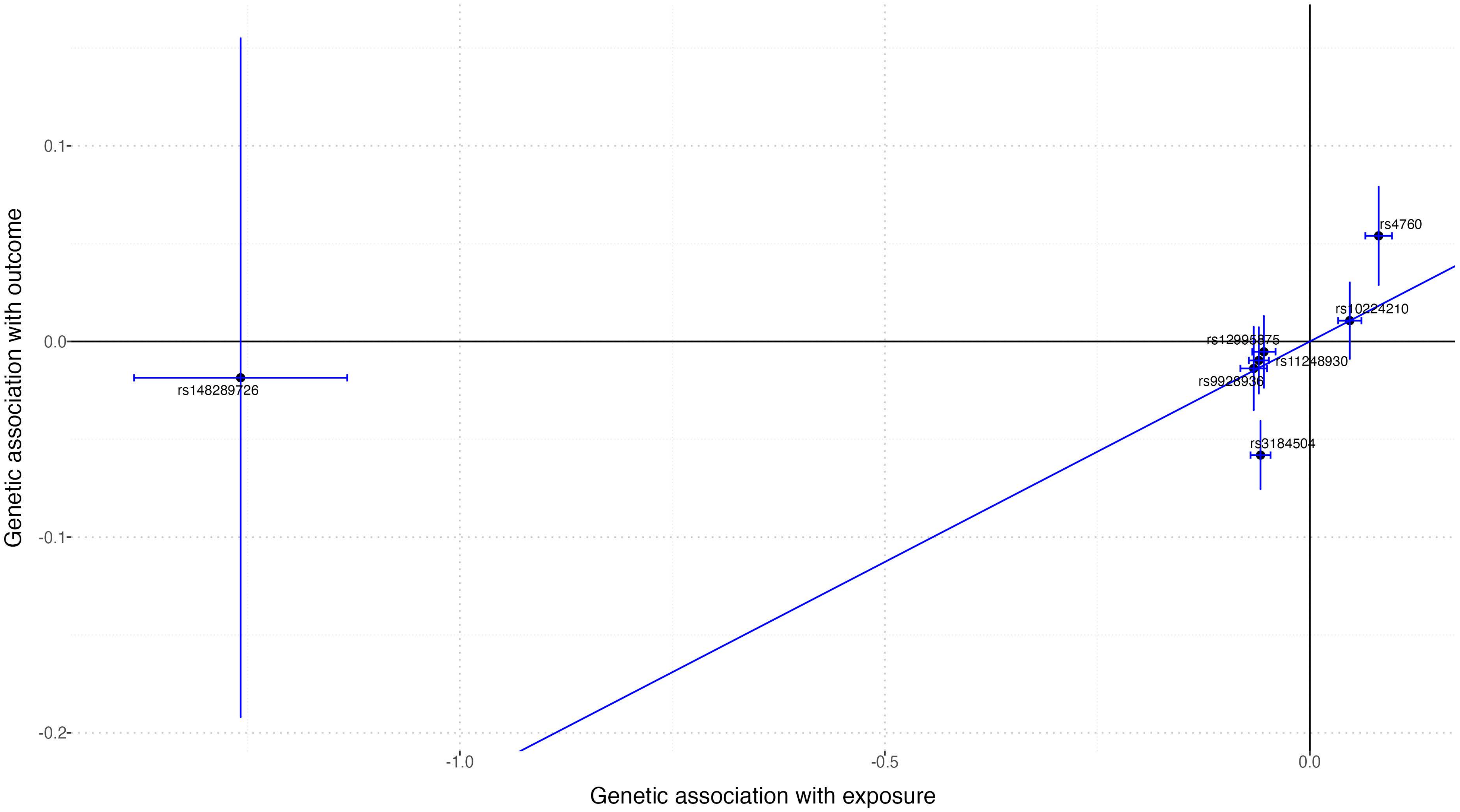

Supplemental Figure 4. Scatter plot showing the association of each SNP with EFNA4 and with IHD in CARDIoGRAMplusC4D.

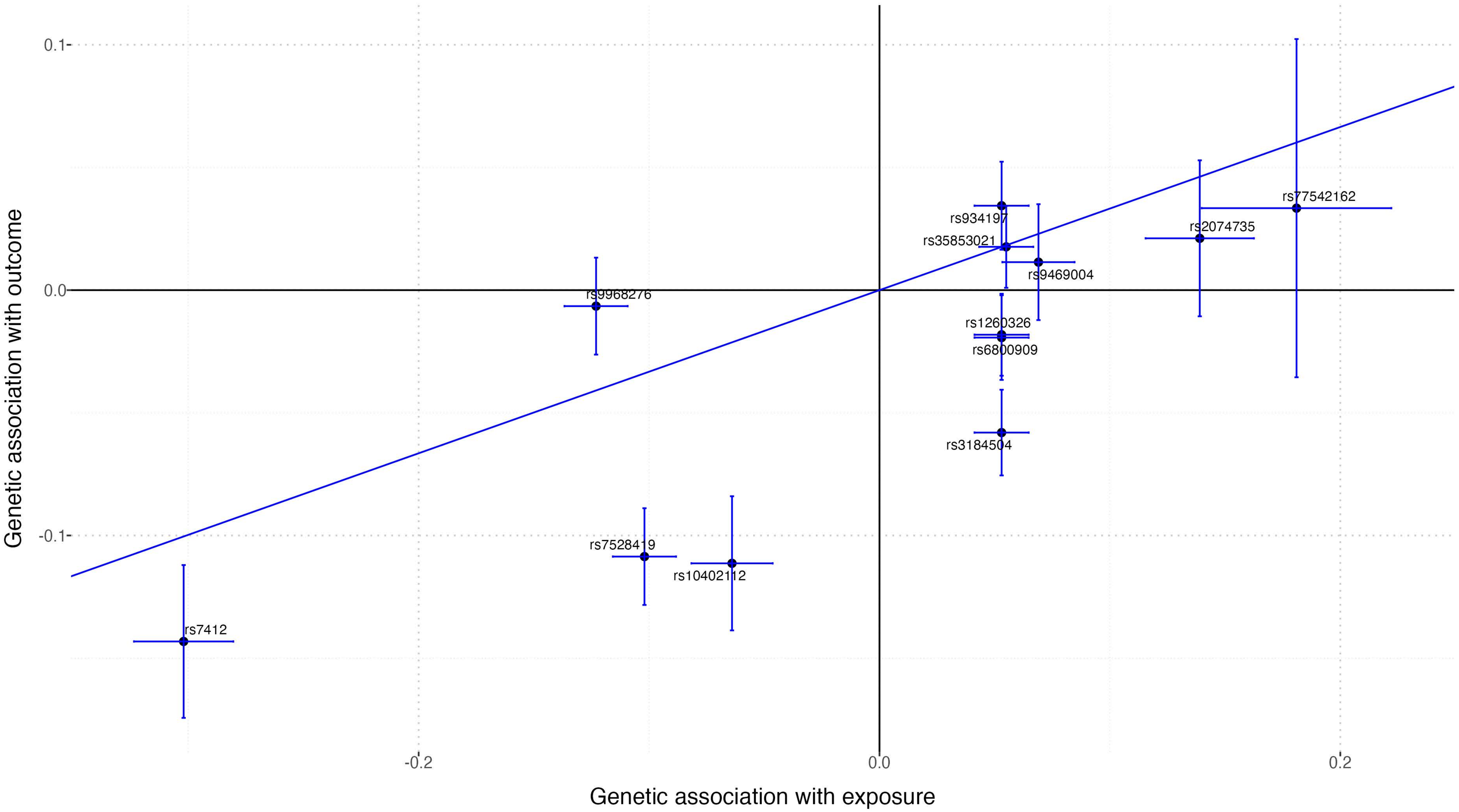

Supplemental Figure 5. Scatter plot showing the association of each SNP with FGFBP1 and with IHD in CARDIoGRAMplusC4D.

a. CARDIoGRAMplusC4D

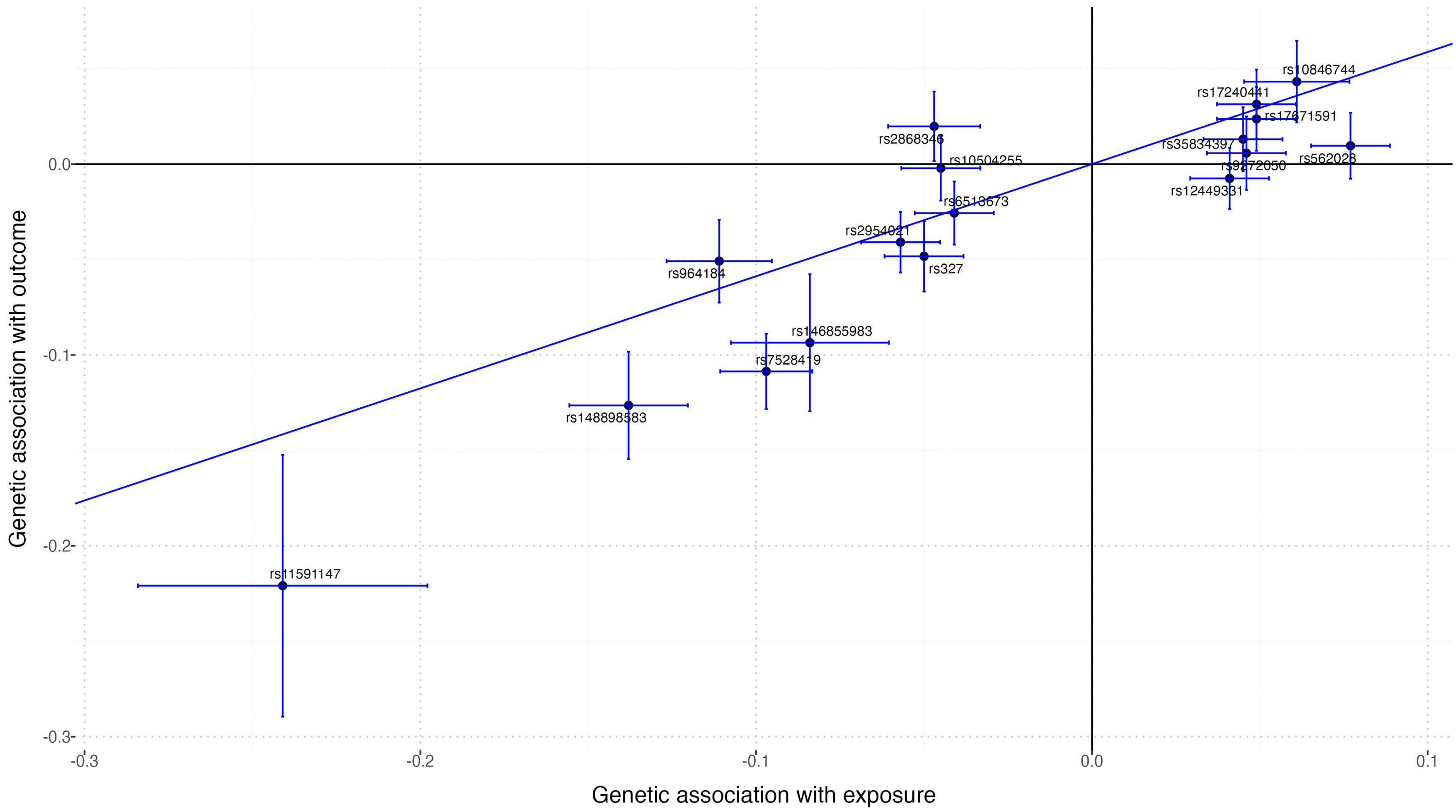

b. FinnGen

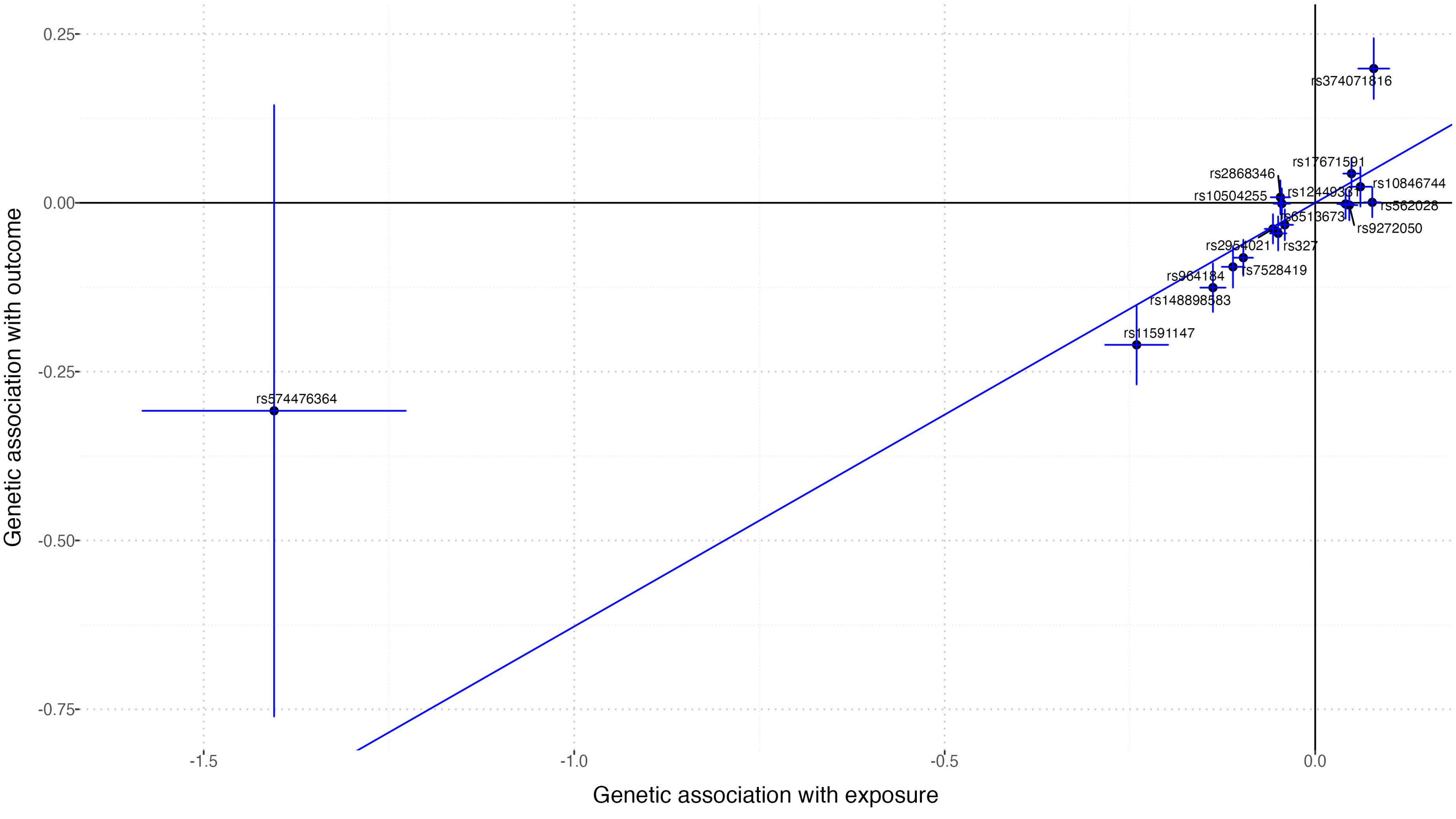

Supplemental Figure 6. Scatter plot showing the association of each SNP with PLA2G7 and with IHD in CARDIoGRAMplusC4D (a) and FinnGen (b).

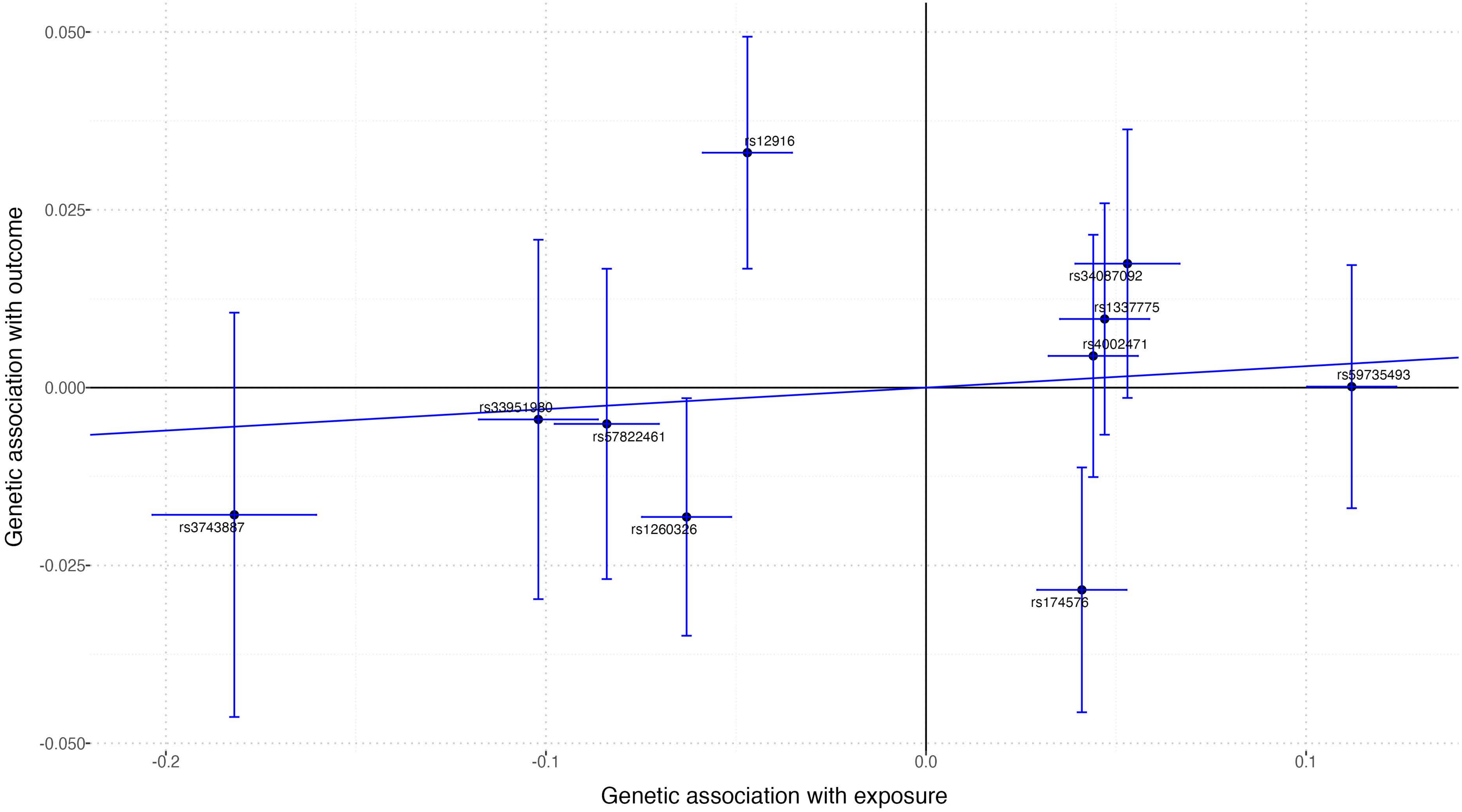

Supplemental Figure 7. Scatter plot showing the association of each SNP with PRSS8 and with IHD in CARDIoGRAMplusC4D.

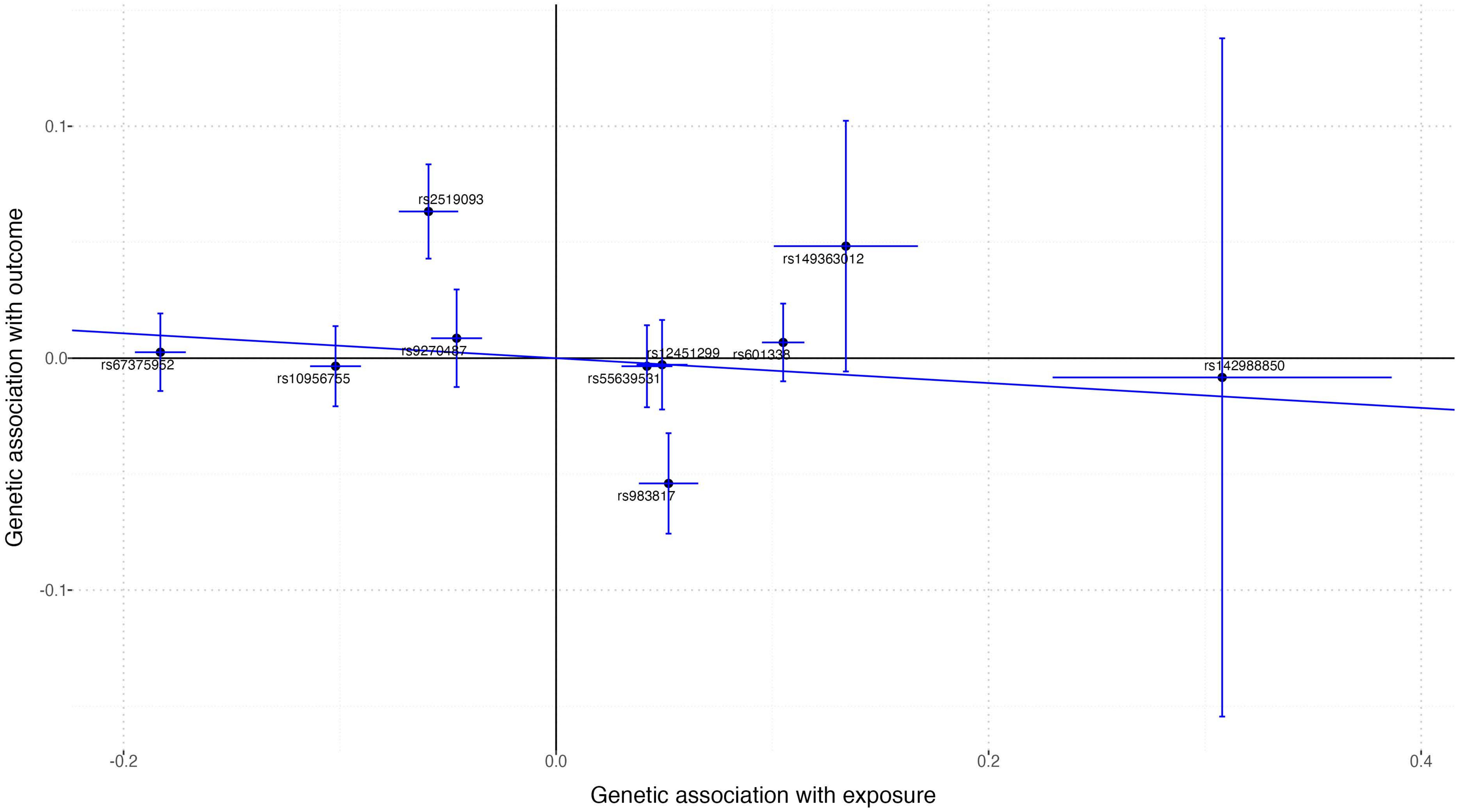

Supplemental Figure 8. Scatter plot showing the association of each SNP with PTPRZ1 and with IHD in CARDIoGRAMplusC4D.

a. CARDIoGRAMplusC4D

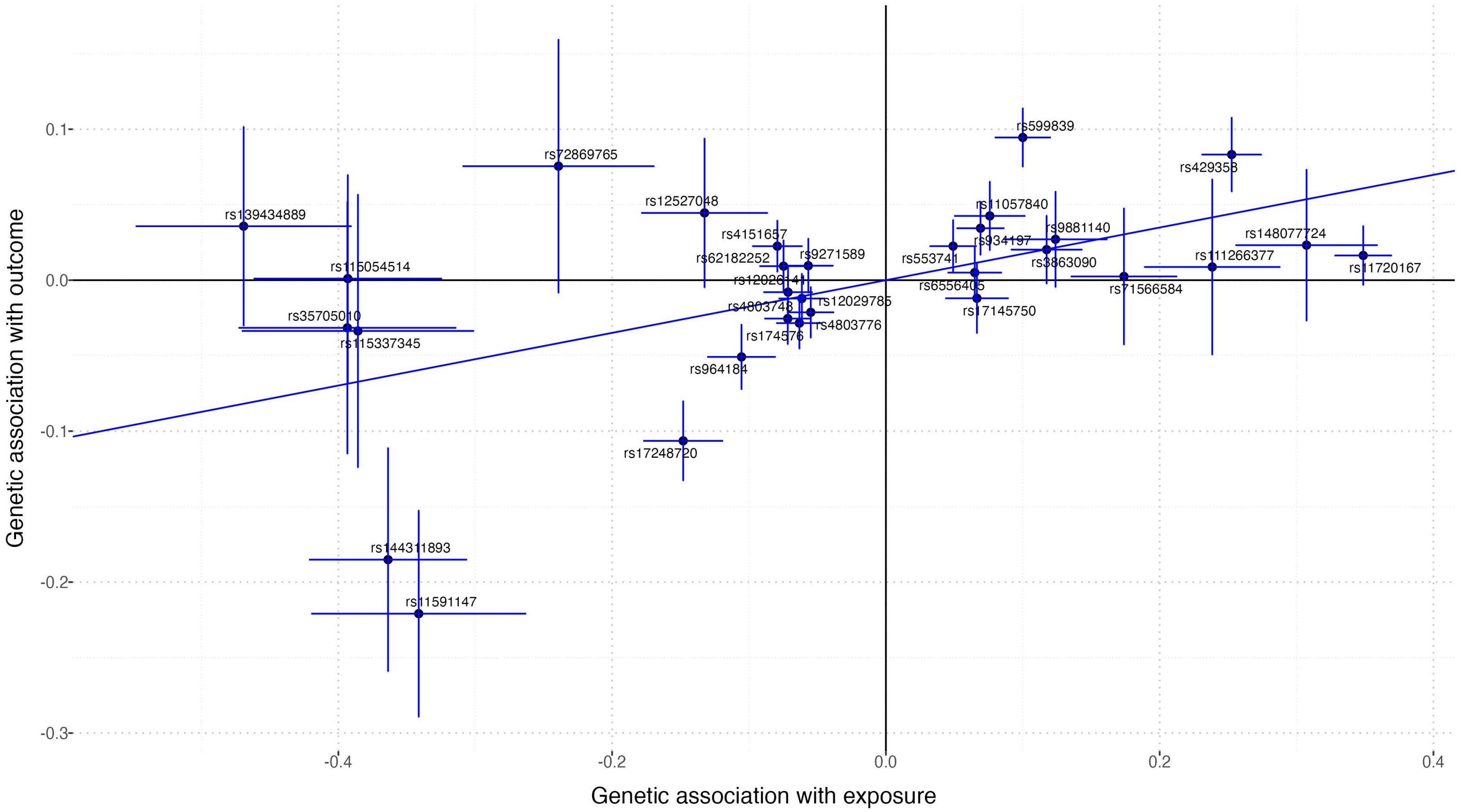

b. FinnGen

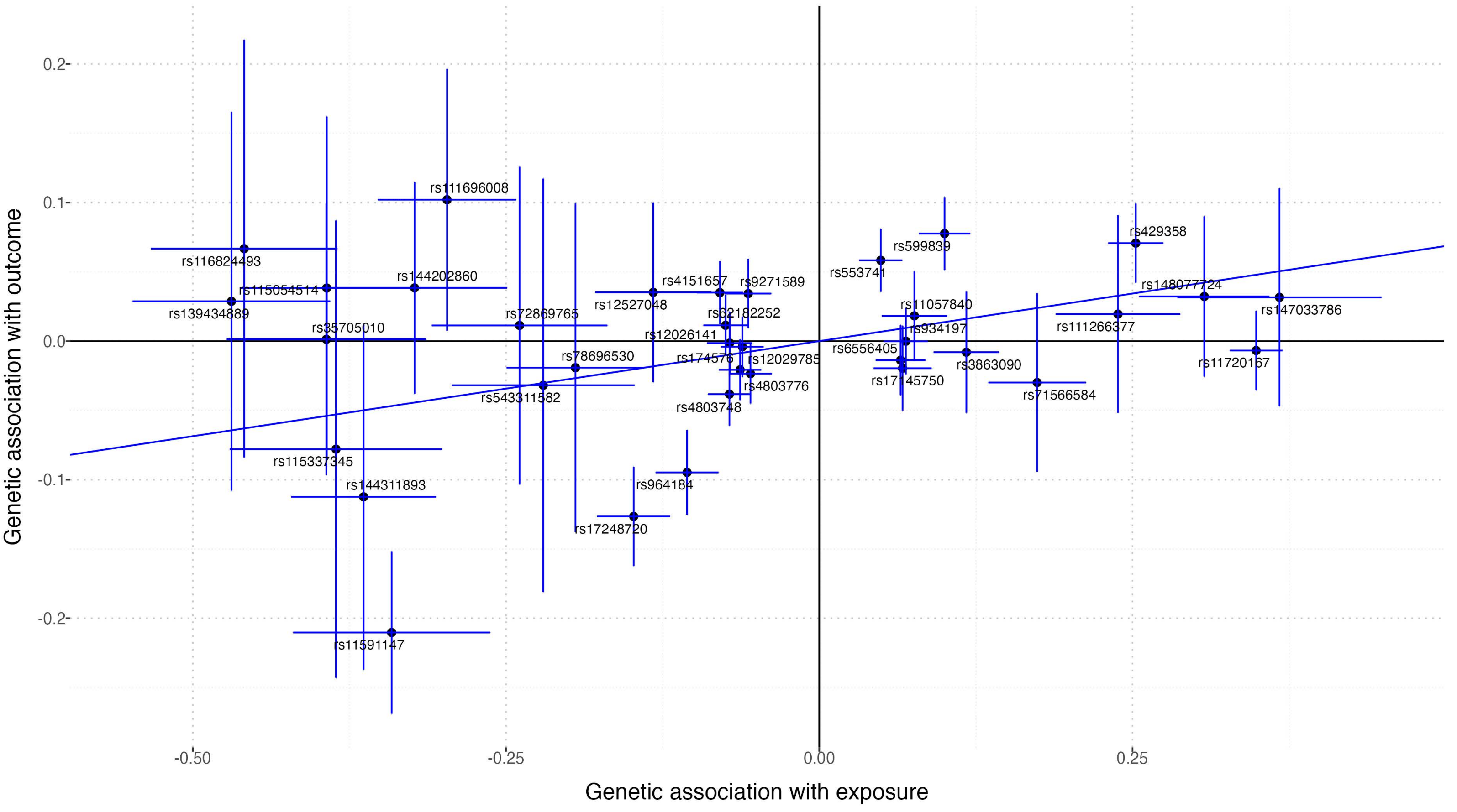

Supplemental Figure 9. Scatter plot showing the association of each SNP with alternative set of genetic instruments for PLA2G7 and with IHD in CARDIoGRAMplusC4D (a) and FinnGen (b).

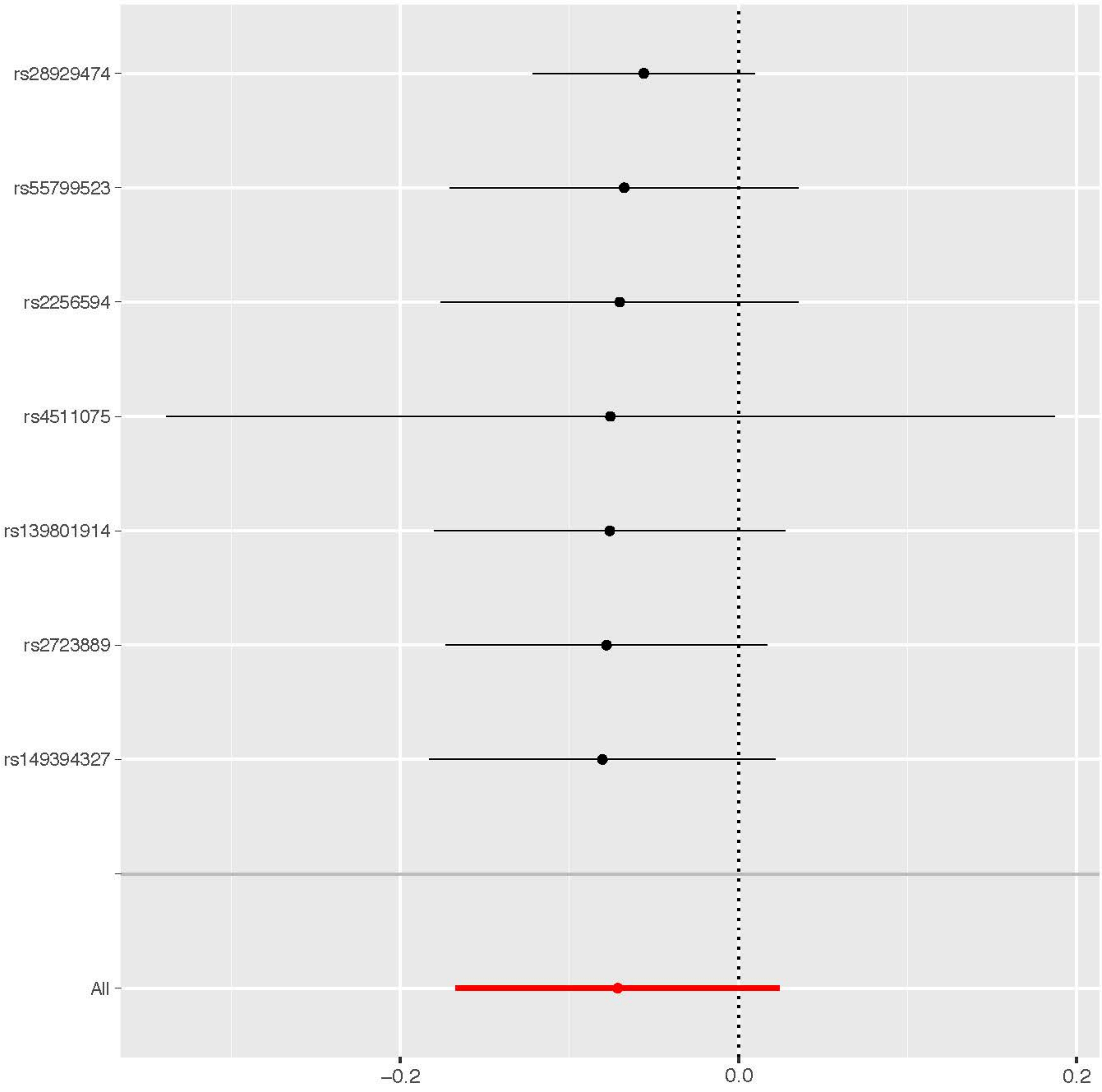

Supplemental Figure 10. Leave-one-out sensitivity analysis on the association of ANGPTL1 and IHD in CARDIoGRAMplusC4D.

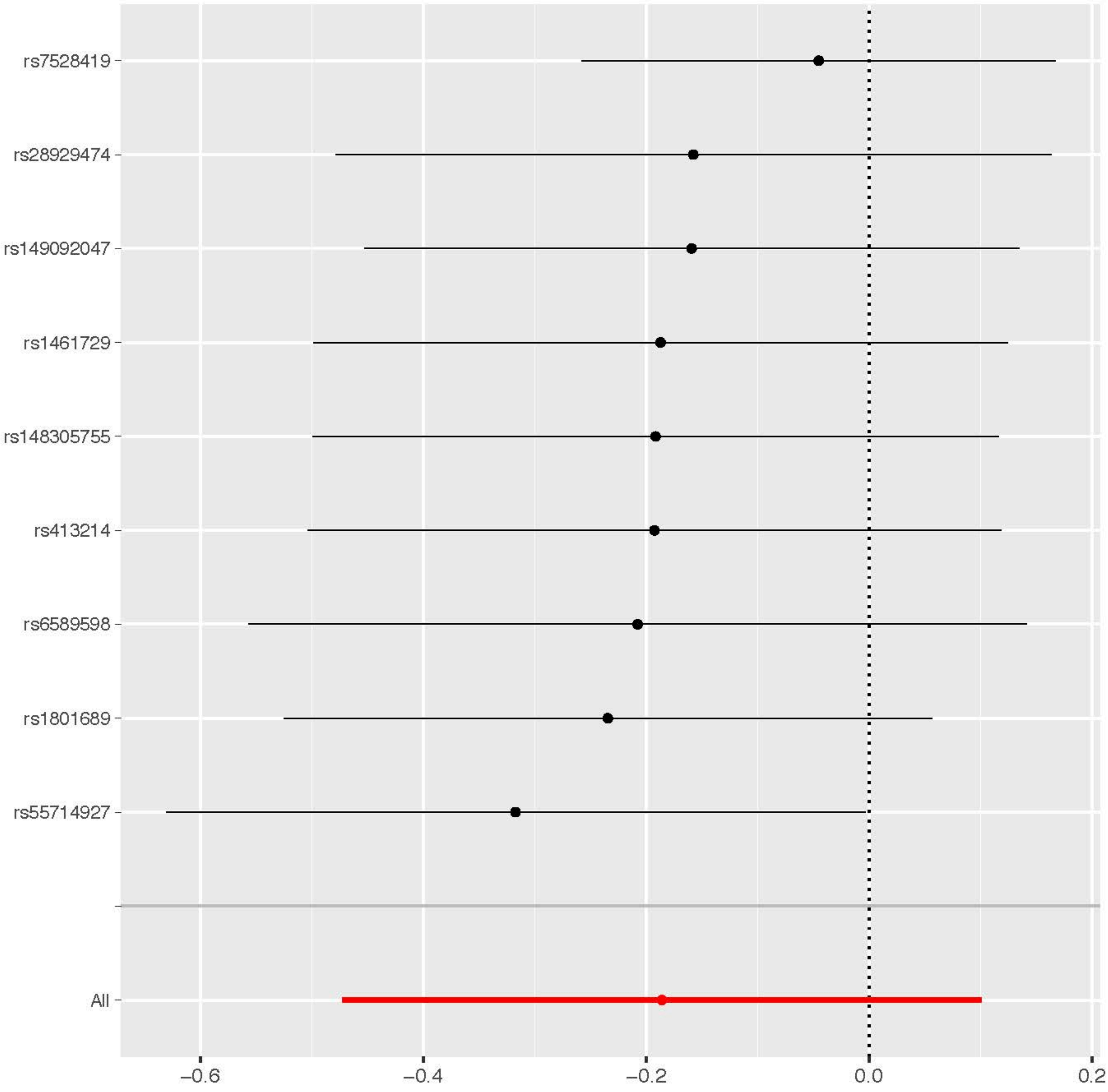

Supplemental Figure 11. Leave-one-out sensitivity analysis on the association of ASGR1 and IHD in CARDIoGRAMplusC4D.

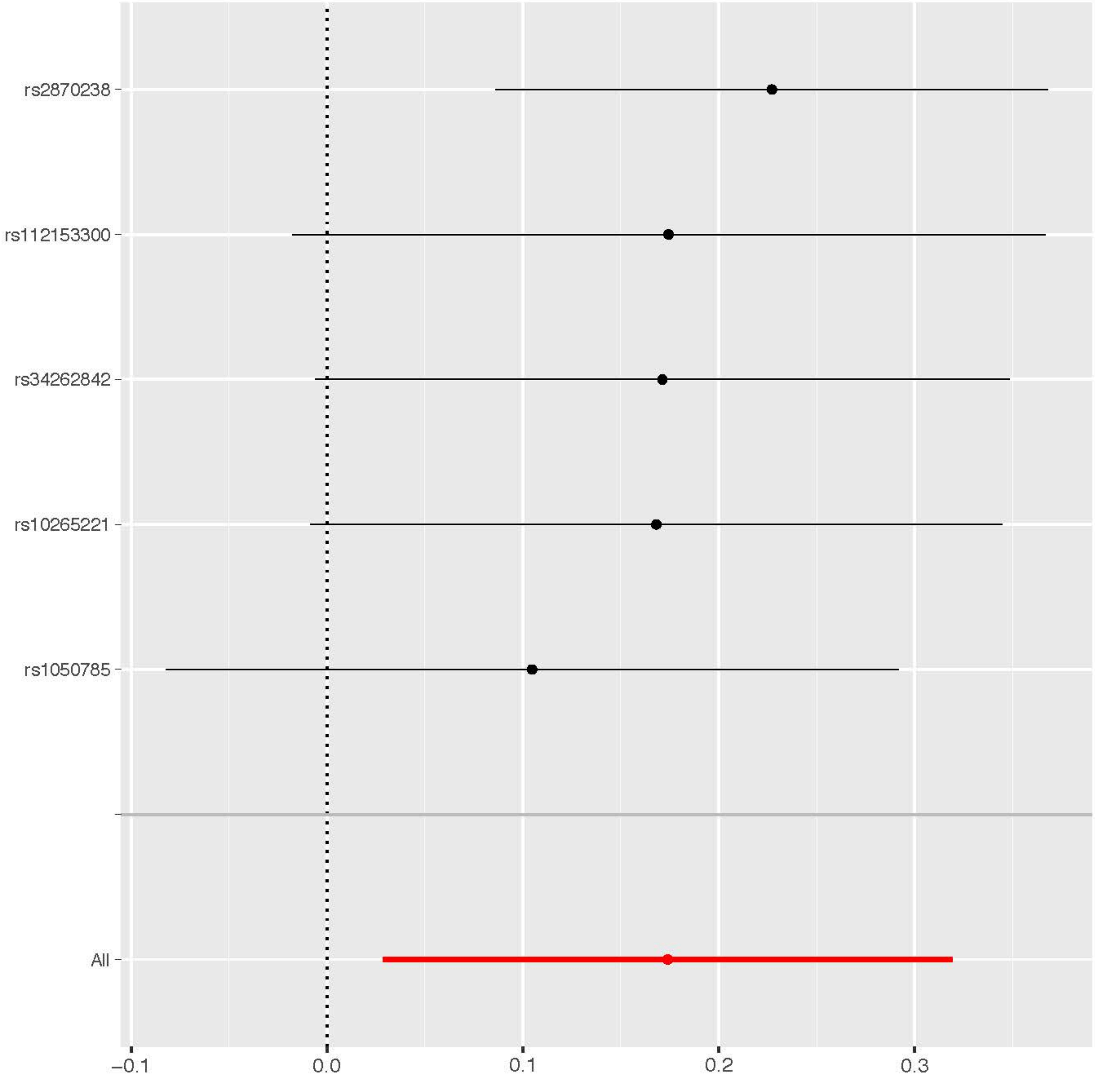

Supplemental Figure 12. Leave-one-out sensitivity analysis on the association of COL6A3 and IHD in CARDIoGRAMplusC4D.

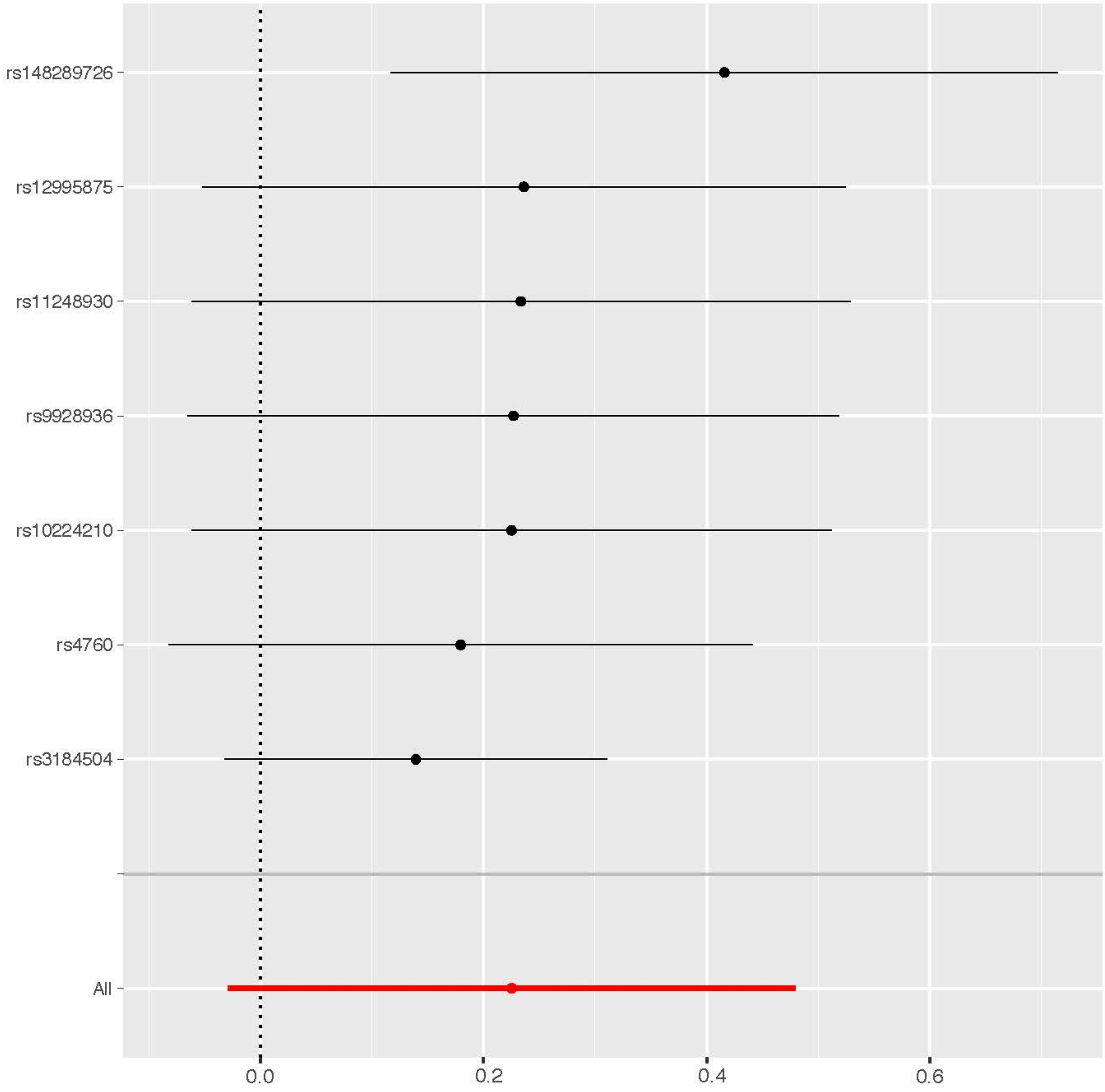

Supplemental Figure 13. Leave-one-out sensitivity analysis on the association of EFNA4 and IHD in CARDIoGRAMplusC4D.

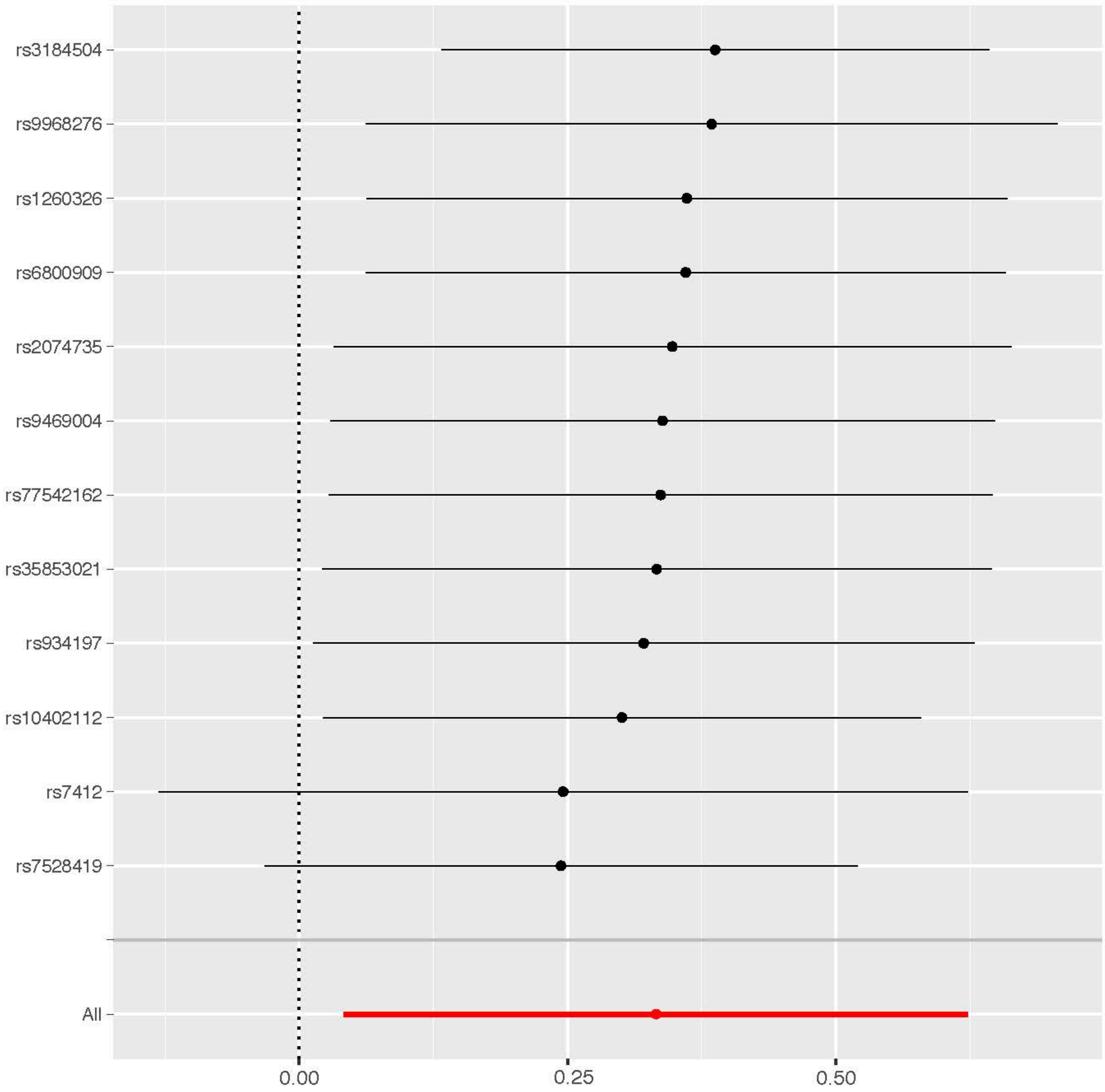

Supplemental Figure 14. Leave-one-out sensitivity analysis on the association of FGFBP1 and IHD in CARDIoGRAMplusC4D.

a. CARDIoGRAMplusC4D

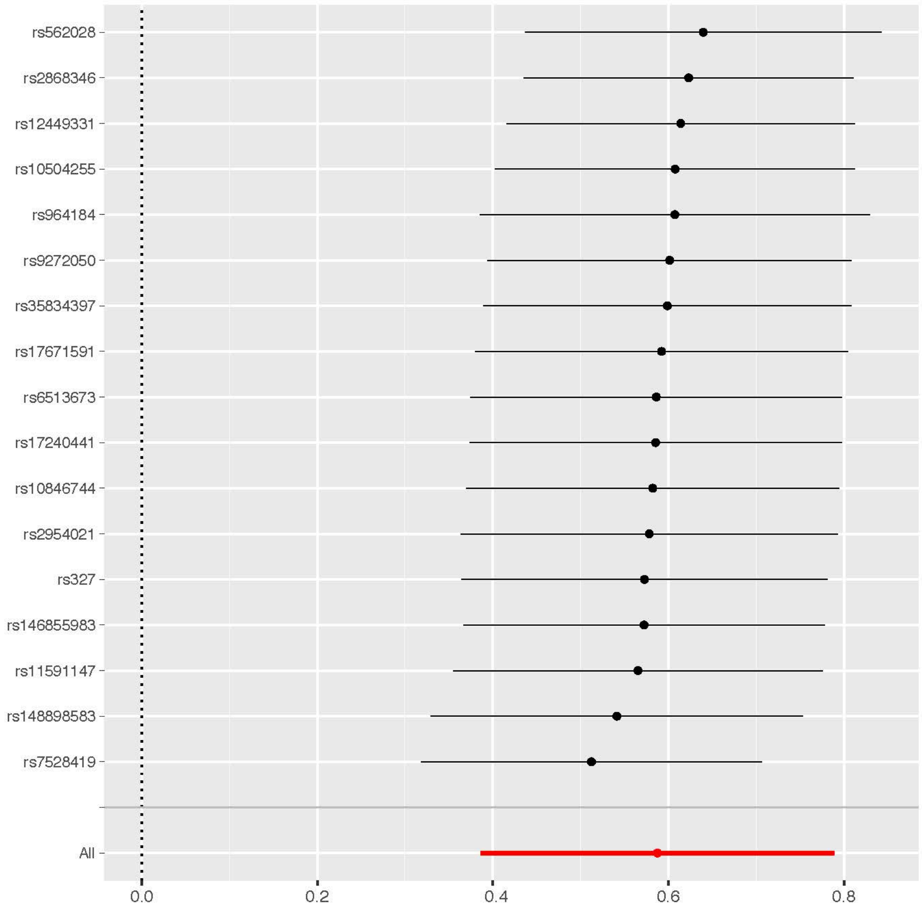

b. FinnGen

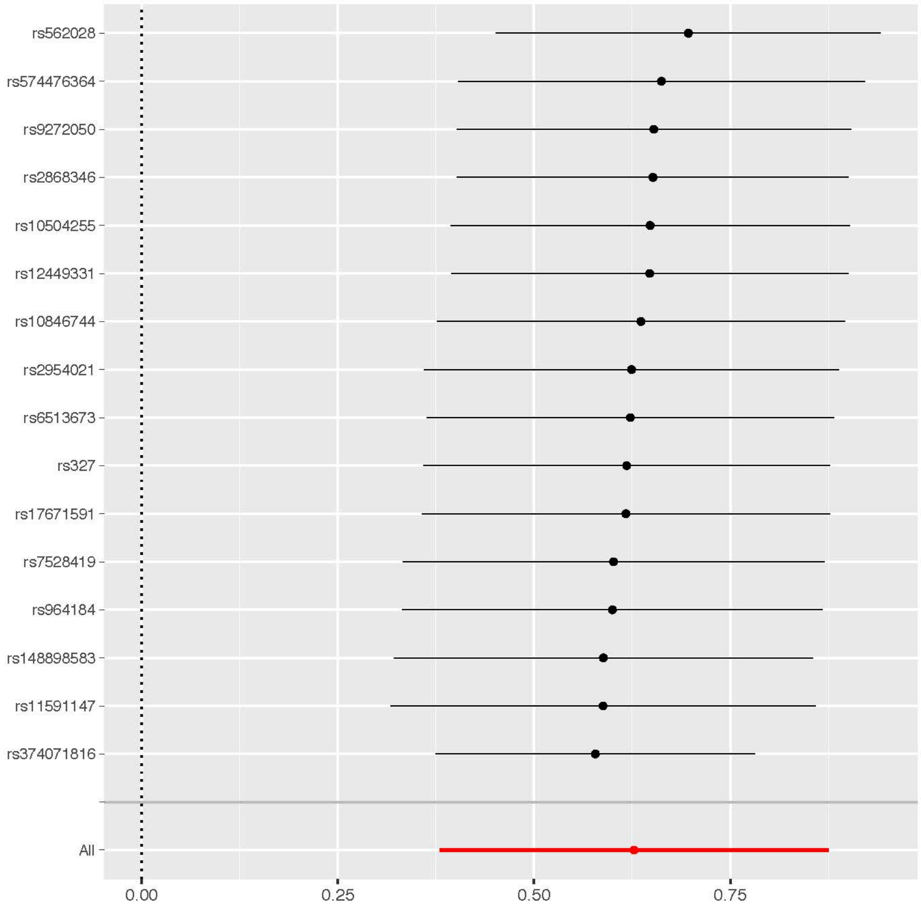

Supplemental Figure 15. Leave-one-out sensitivity analysis on the association of PLA2G7 and IHD in CARDIoGRAMplusC4D (a) and FinnGen (b).

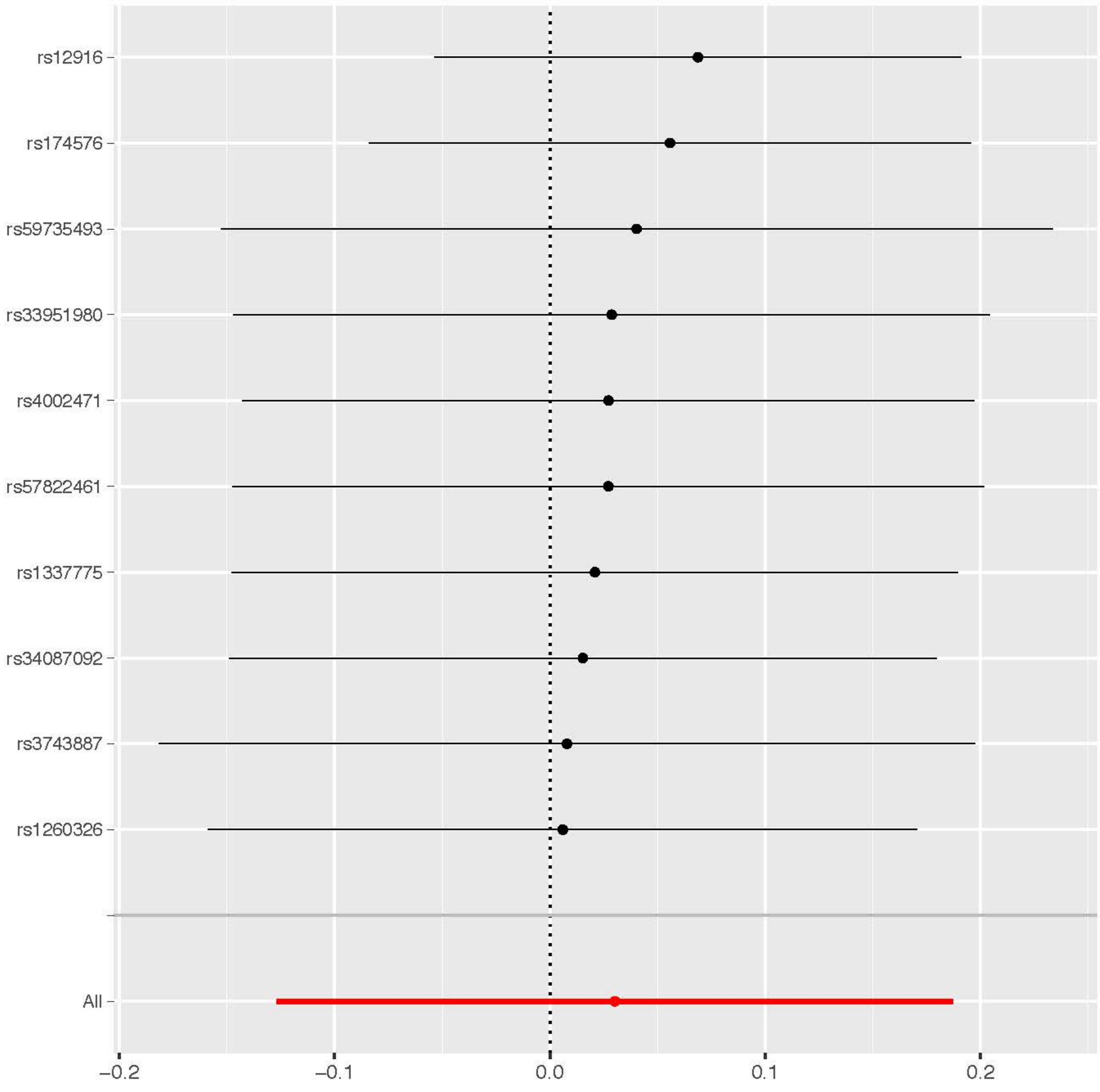

Supplemental Figure 16. Leave-one-out sensitivity analysis on the association of PRSS8 and IHD in CARDIoGRAMplusC4D.

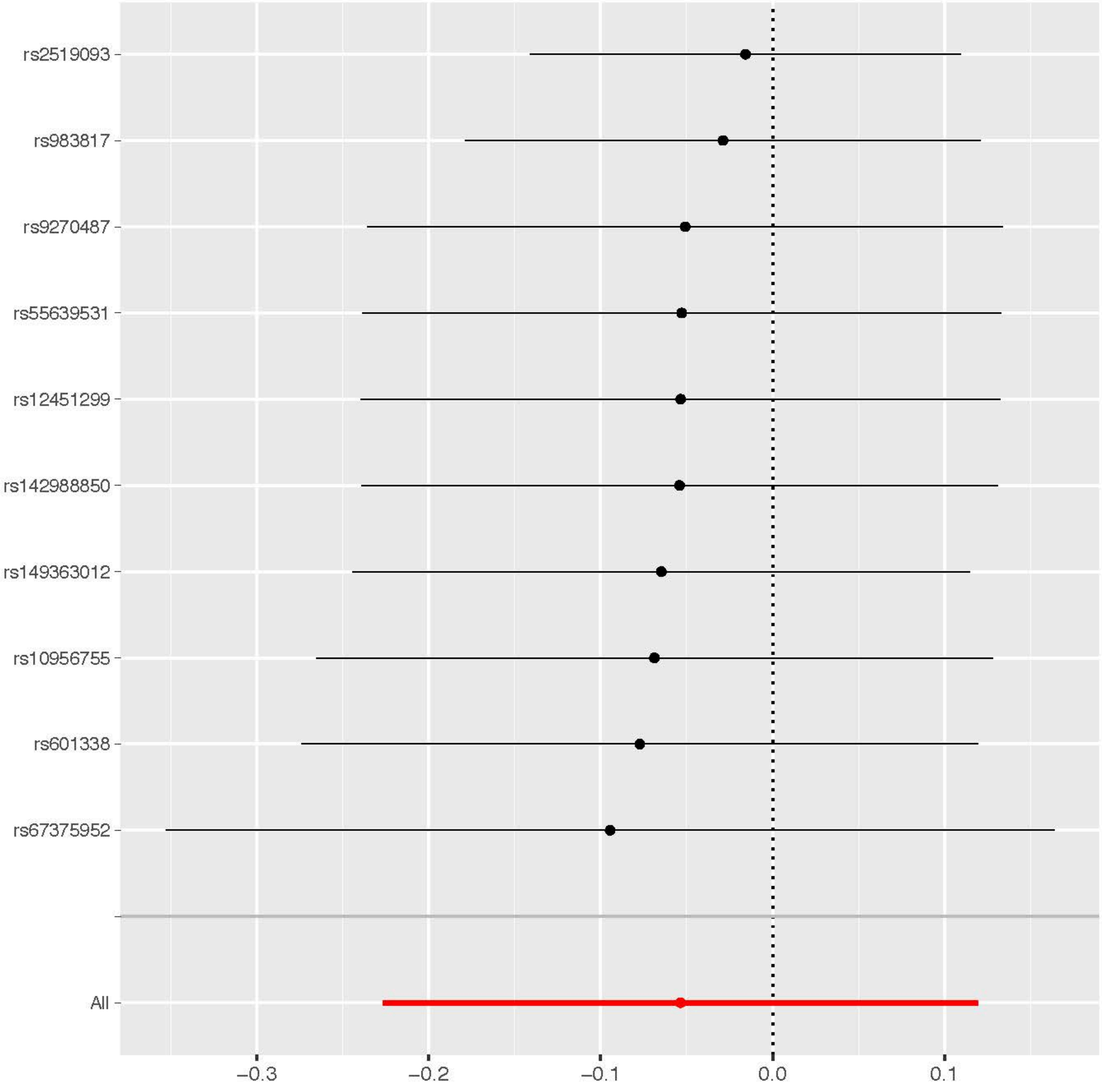

Supplemental Figure 17. Leave-one-out sensitivity analysis on the association of PTPRZ1 and IHD in CARDIoGRAMplusC4D.

a. CARDIoGRAMplusC4D

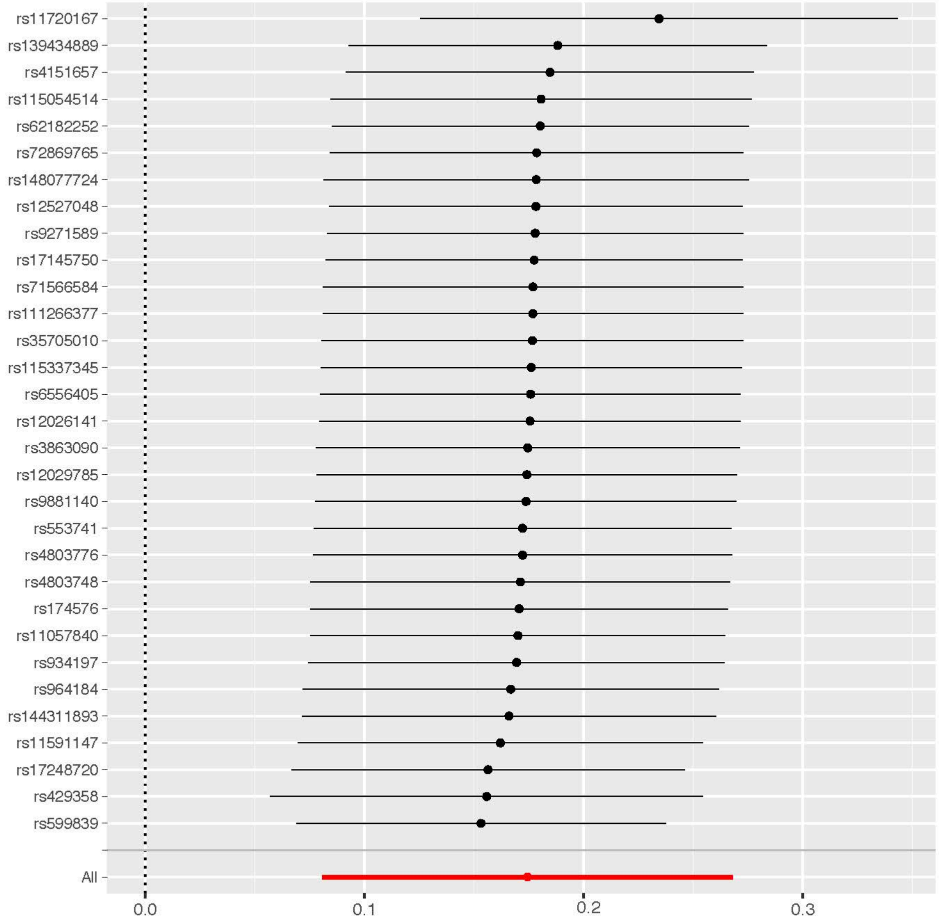

b. FinnGen

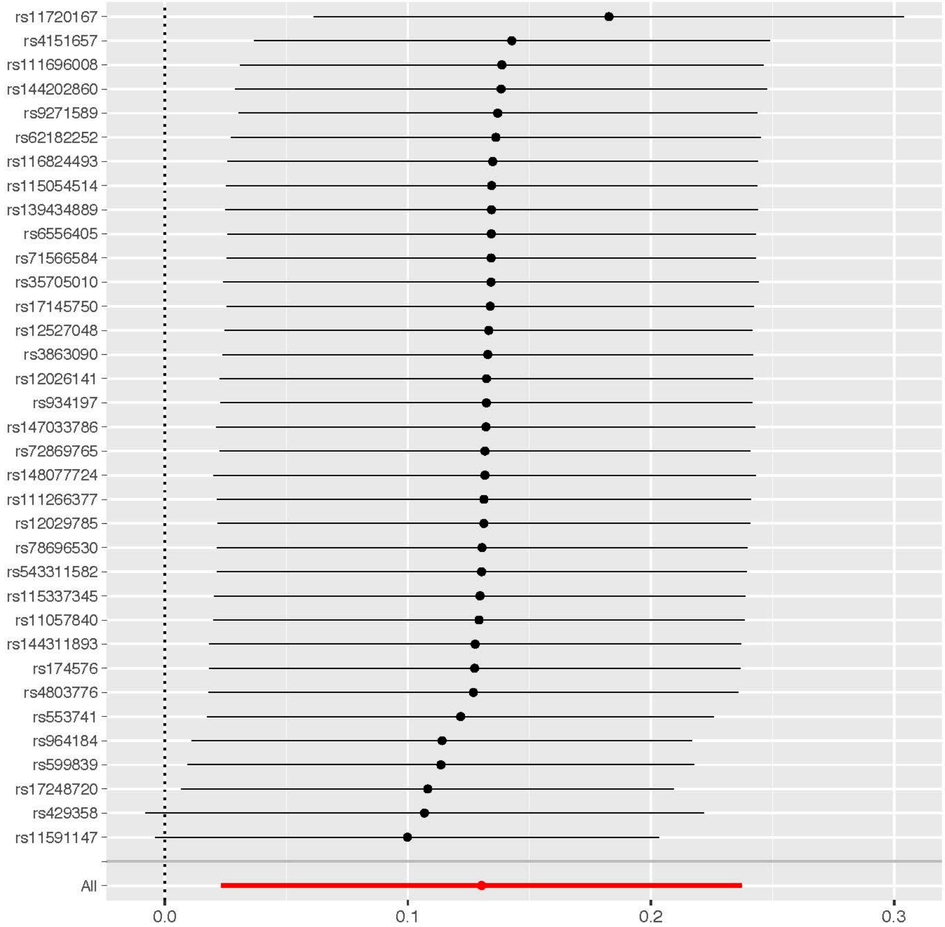

Supplemental Figure 18. Leave-one-out sensitivity analysis on the association of alternative set of genetic instruments for PLA2G7 and IHD in CARDIoGRAMplusC4D (a) and FinnGen (b).

a. DIAGRAM

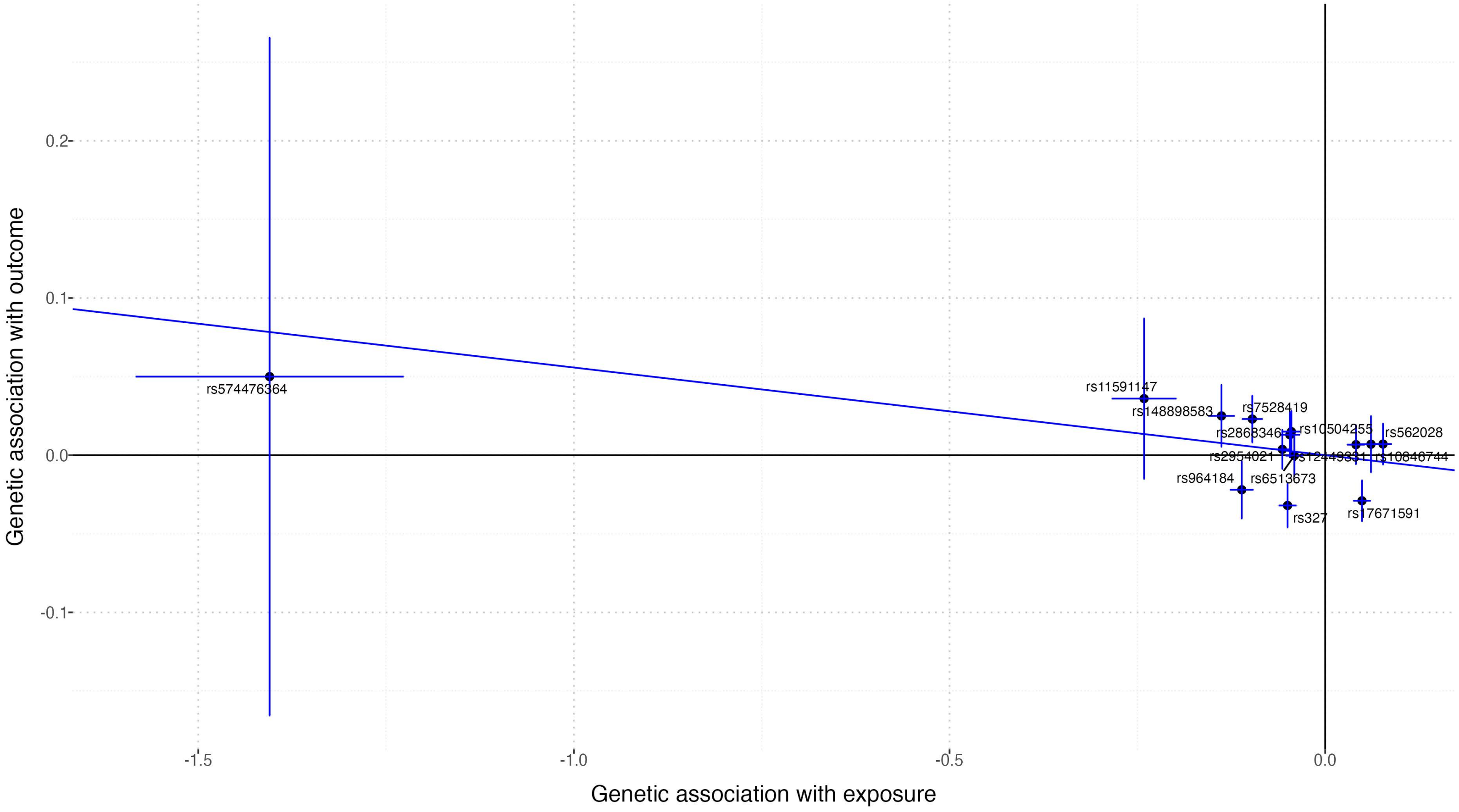

b. FinnGen

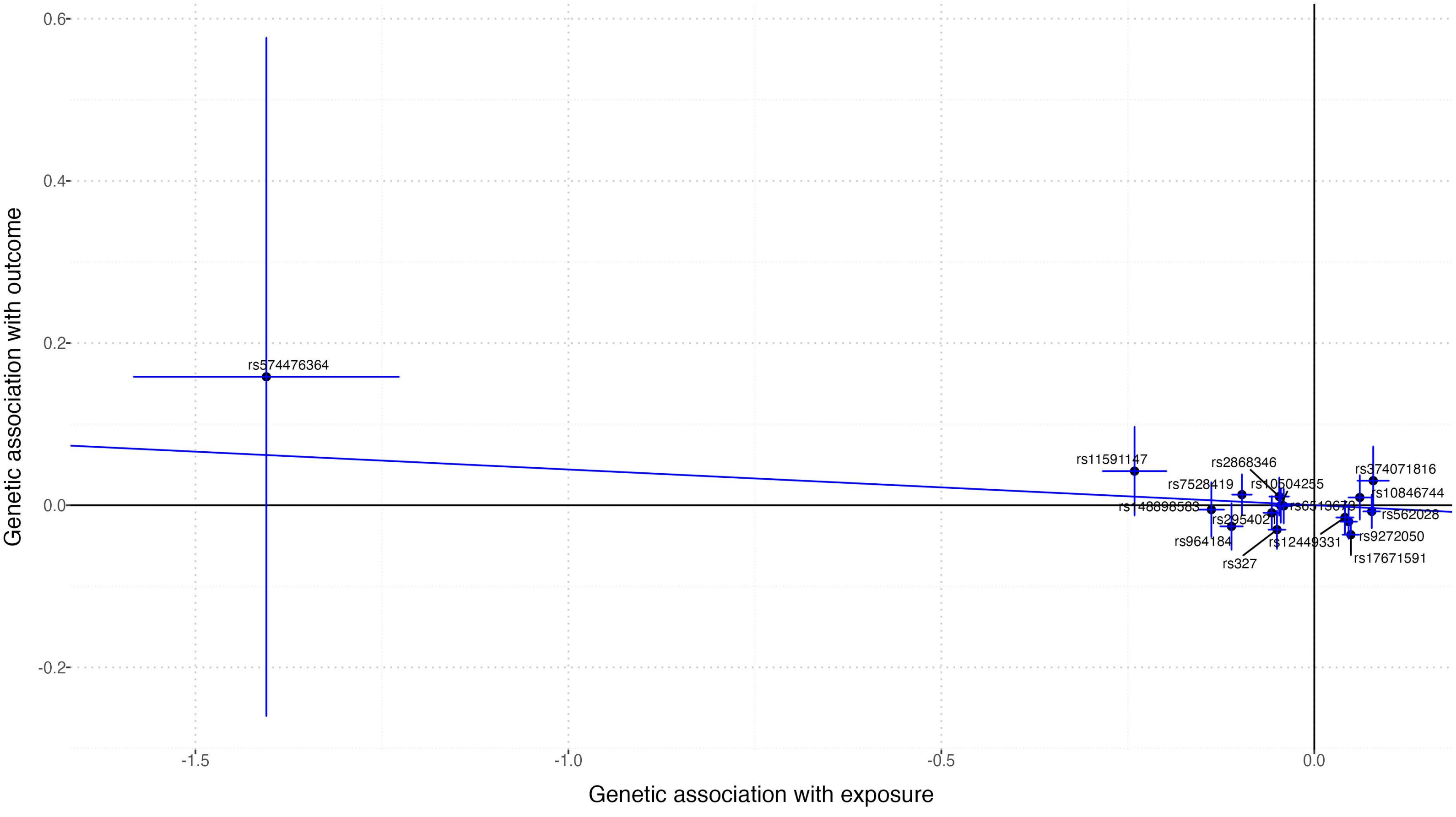

Supplemental Figure 19. Scatter plot showing the association of each SNP with PLA2G7 and with T2D in DIAGRAM (a) and in FinnGen (b).

a. CKD

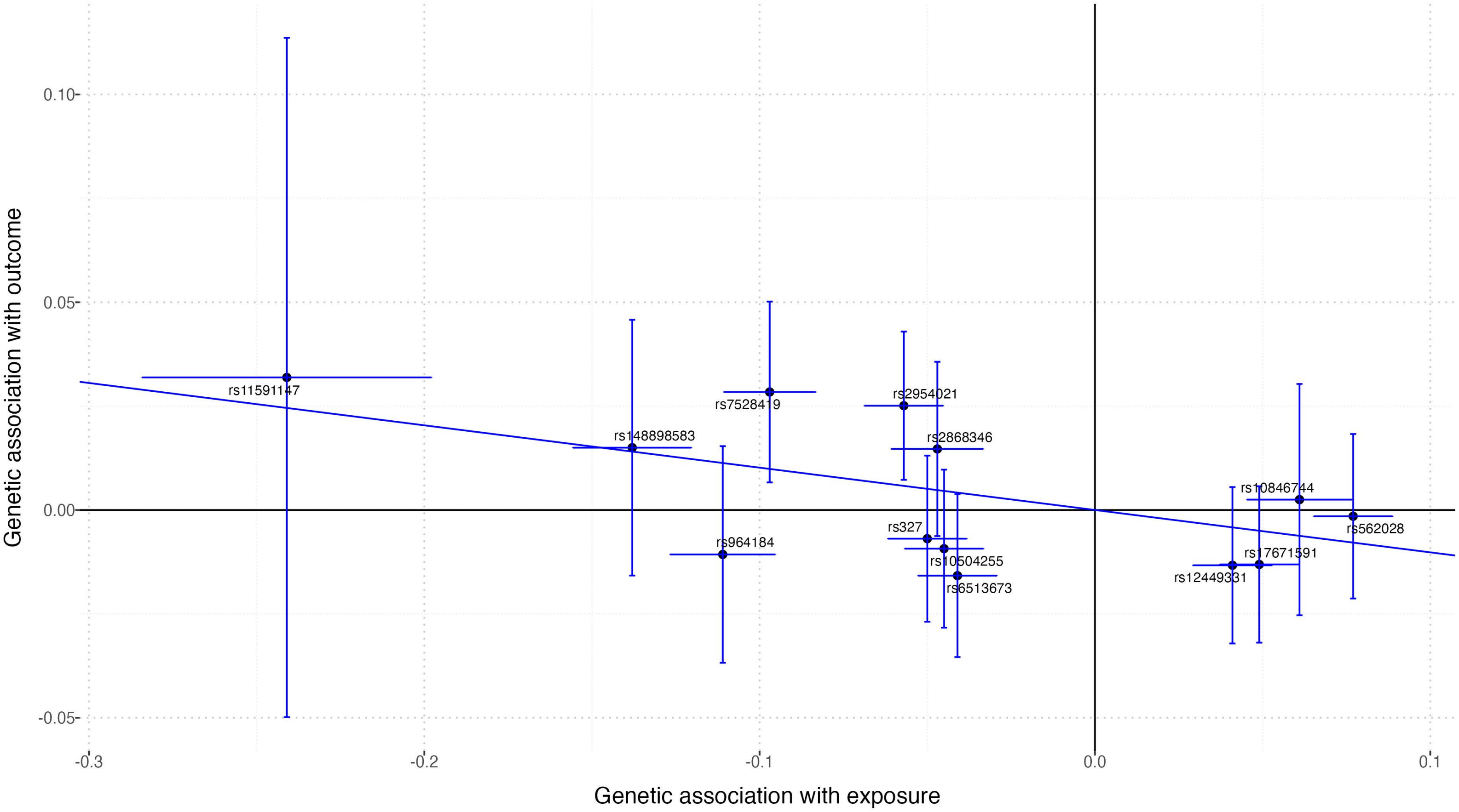

b. eGFR

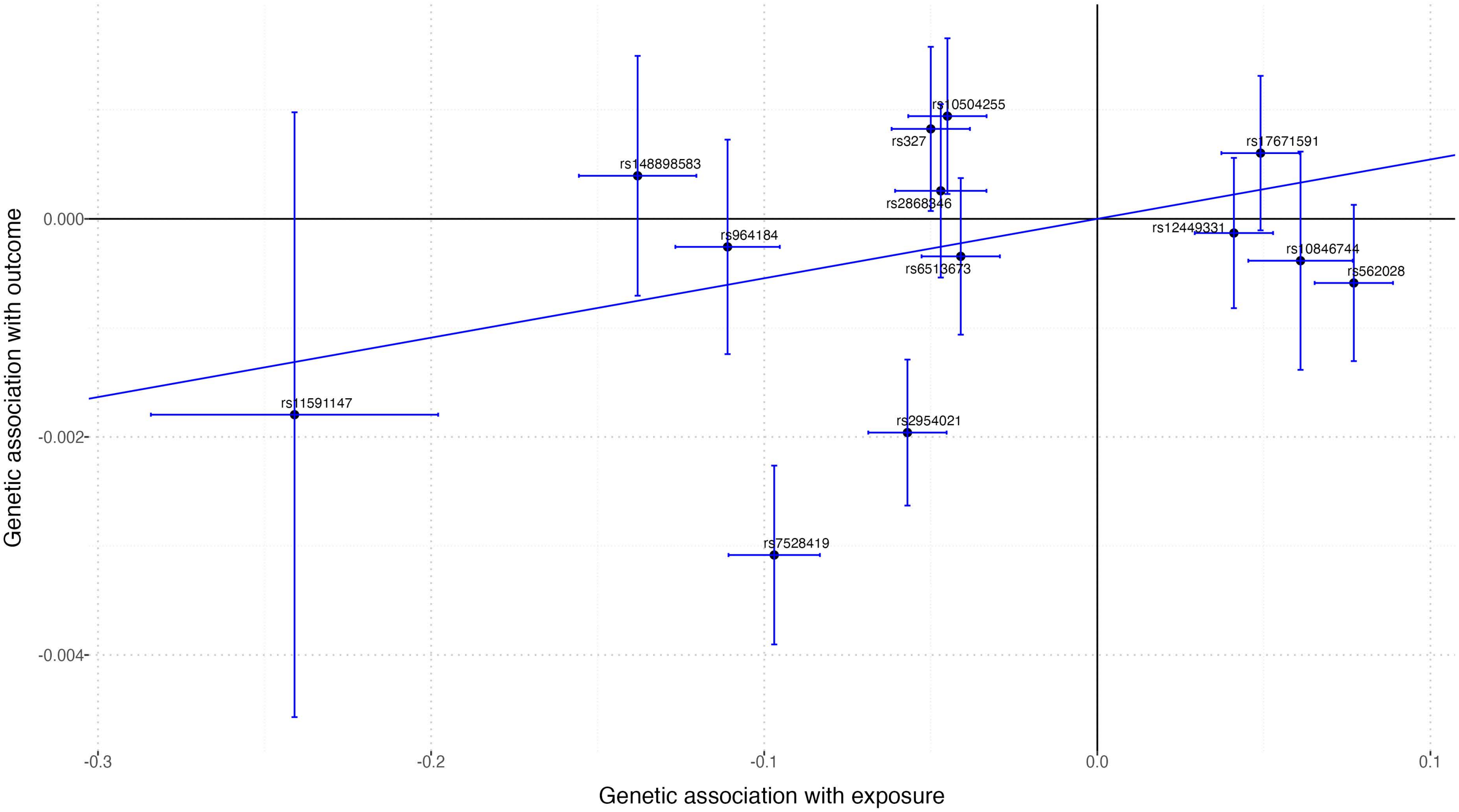

Supplemental Figure 20. Scatter plot showing the association of each SNP with PLA2G7 and with CKD in CKDGen (a), and eGFR in CKDGen (b).

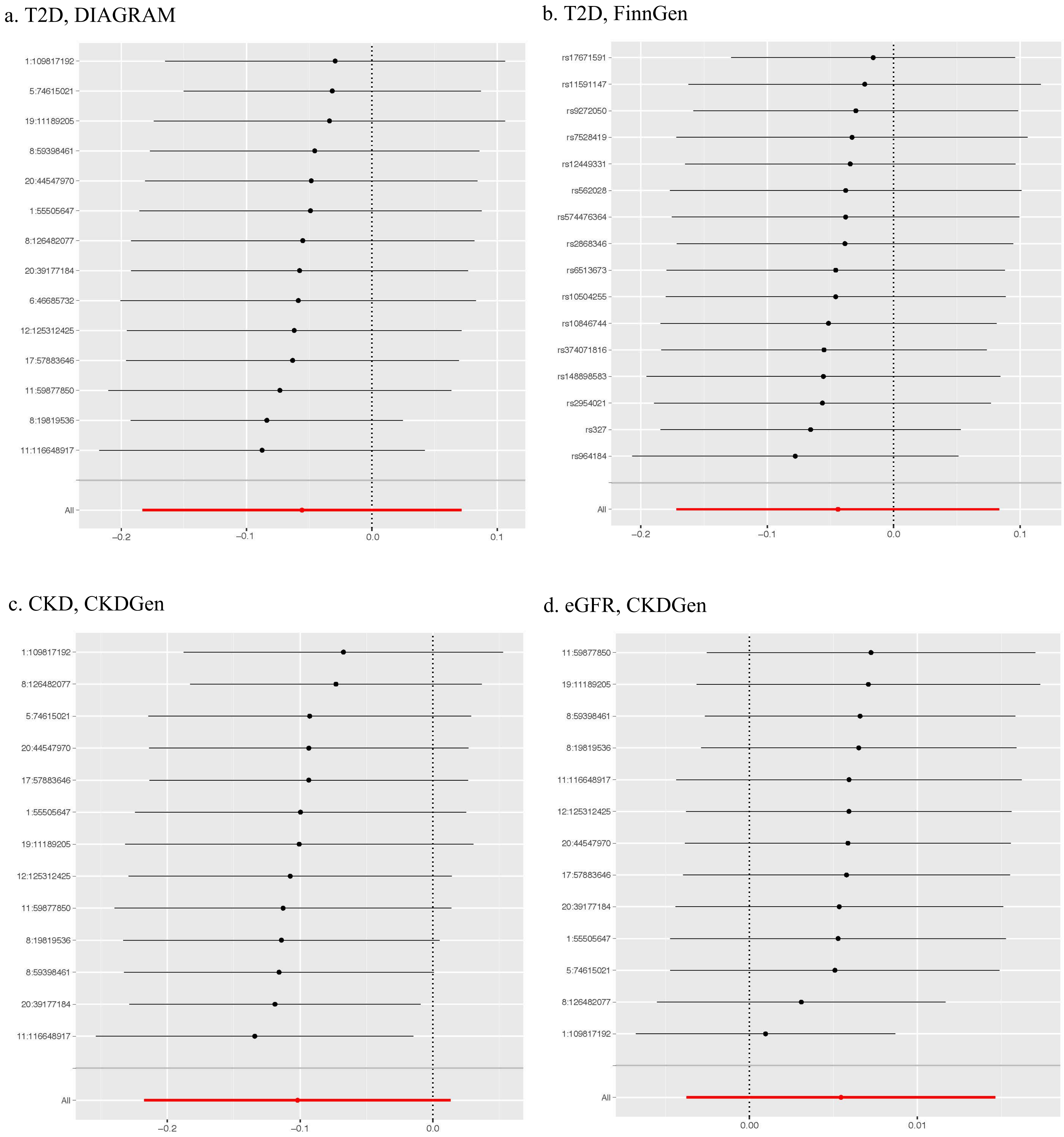

Supplemental Figure 21. Leave-one-out sensitivity analysis on the association of PLA2G7 and T2D in DIAGRAM (a), T2D in FinnGen (b), CKD in CKDGen (c), and eGFR in CKDGen (d).

Supplemental Table 1. Overview of genome-wide association studies incorporated in this study

Outcomes	Information of genome-wide association study (GWAS)			
	Data sources	Sample size	Covariates	Reference
Primary outcomes				
IHD	CARDIoGRAMplusC4D	76,014 cases, 264,785 controls	assay array and the first five principal components provided by UK Biobank	(Nelson, Goel et al. 2017)
	FinnGen	30,952 cases, 187,840 controls	Age, sex, genotyping batch, the first 10 principal components	https://www.finngen.fi/en
Secondary outcomes				
T2D	DIAGRAM	74,124 cases, 824,006 controls	Sex, study-specific covariates, population structure and relatedness	(Mahajan, Taliun et al. 2018)
T2D	FinnGen	32,469 cases, 183,185 controls	Age, sex, genotyping batch, the first 10 principal components	https://www.finngen.fi/en
CKD	CKDGen	41,395 cases, 439,303 controls	Age, sex, study site, genetic principal	(Wuttke, Li et al. 2019)
eGFR	CKDGen	n = 567,460	components, relatedness and other study-specific features	

IHD, Ischemic Heart Disease; T2D, Type 2 Diabetes; CKD, Chronic Kidney Disease; eGFR, Estimated Glomerular Filtration Rate; CARDIoGRAMplusC4D, "Coronary ARtery DIsease Genome-wide Replication and Meta-analysis (CARDIoGRAM) plus The Coronary Artery Disease (C4D) Genetics" consortium; DIAGRAM, "Diabetes Genetics Replication and Meta-analysis" consortium; CKDGen: Chronic Kidney Disease Genetics Consortium

Supplemental Table 2. Genetic instruments for the 9 proteins affected by statins

SNP*	chr	effect allele	other allele	beta	se	-log(p-value)†	F statistics
**ANGPTL1**							
rs4511075	1	A	G	-0.28	0.01	459.2	2209.0
rs2256594	6	G	A	0.06	0.01	14.5	58.1
9:101562826	9	C	CT	-0.08	0.01	14.9	70.6
rs12359178	10	A	G	0.07	0.01	14.5	55.4
rs745680717	12	C	CCTG	0.05	0.01	17.6	78.0
rs2723889	12	C	T	0.04	0.01	10.8	49.0
rs28929474	14	T	C	0.20	0.02	21	93.4
rs200293726	16	T	A	0.05	0.01	12.3	61.4
rs186021206	17	A	G	0.37	0.04	18.3	78.9
rs149394327	17	C	G	0.26	0.02	49.3	228.5
rs55799523	19	A	C	0.05	0.01	15.1	59.5
**ASGR1**							
rs7528419	1	G	A	0.12	0.01	61.4	289.0
rs62323607	4	T	C	0.05	0.01	12.2	58.8
rs413214	5	A	G	0.05	0.01	13.9	61.4
rs1461729	8	G	A	-0.07	0.01	12	50.4
rs782134971	9	G	GAAACTGCC	-0.05	0.01	13.5	55.2
rs6589598	11	G	A	-0.14	0.01	74.1	377.5
rs148305755	13	A	G	0.12	0.02	12.3	52.3
rs28929474	14	T	C	0.33	0.02	53.2	227.7
rs55714927	17	T	C	-0.19	0.01	132.7	552.3
rs1801689	17	C	A	0.19	0.02	27.4	115.0
**COL6A3**							
rs1050785	2	A	C	0.09	0.01	47.7	225.0
rs2870238	4	T	C	0.04	0.01	13.1	53.8
rs10265221	7	C	T	0.05	0.01	12.6	47.0
rs34262842	16	G	A	-0.06	0.01	10.8	47.3
rs186021206	17	A	G	0.38	0.04	19	81.9
rs112153300	21	A	G	-0.11	0.01	27	127.7
**EFNA4**							
rs148289726	1	T	C	-1.26	0.06	85.7	386.4
rs12995875	2	C	T	-0.05	0.01	14.6	59.5
rs10224210	7	C	T	0.05	0.01	11.5	45.1
rs3184504	12	C	T	-0.06	0.01	20.9	93.4
rs11248930	16	T	C	-0.06	0.01	23.1	100.0
rs9928936	16	A	G	-0.07	0.01	16.5	68.1
rs4760	19	G	A	0.08	0.01	21.8	102.5
**FGFBP1**							
rs7528419	1	G	A	-0.10	0.01	47.4	212.3
rs934197	2	A	G	0.05	0.01	16.2	78.0
rs1260326	2	C	T	0.05	0.01	17.6	78.0
rs6800909	3	G	T	0.05	0.01	15.8	78.0
rs9968276	4	A	G	-0.12	0.01	62.2	308.8
rs9469004	6	C	T	0.07	0.01	17.4	74.4
rs3184504	12	C	T	0.05	0.01	18.1	78.0
rs35853021	15	T	G	0.06	0.01	18.7	84.0

rs77542162	17	G	A	0.18	0.02	18	74.3
rs10402112	19	A	T	-0.06	0.01	12.3	50.6
rs7412	19	T	C	-0.30	0.01	178.6	753.8
rs2074735	22	C	G	0.14	0.01	32	134.2
**PCOLCE**							
rs4548095	7	T	C	0.17	0.01	63.7	285.6
**PLA2G7**							
rs11591147	1	T	G	-0.24	0.02	26.4	120.0
1:63159212	1	T	TA	0.05	0.01	13.3	56.3
rs7528419	1	G	A	-0.10	0.01	45.1	192.0
2:21266774	2	G	GGCAGCGCCA	0.05	0.01	15.3	66.7
rs765299456	2	G	GTTA	-0.08	0.01	11.8	49.0
rs17671591	5	T	C	0.05	0.01	15.8	66.7
rs9272050	6	A	G	0.05	0.01	13.9	58.8
rs574476364	6	T	C	-1.41	0.09	53.3	238.4
rs374071816	6	A	T	0.08	0.01	13	51.6
rs327	8	G	T	-0.05	0.01	14.3	69.4
rs10504255	8	A	G	-0.05	0.01	12.7	56.3
rs2954021	8	G	A	-0.06	0.01	22.4	90.3
rs562028	11	C	A	0.08	0.01	36.4	164.7
rs964184	11	C	G	-0.11	0.01	41.1	192.5
rs10846744	12	C	G	0.06	0.01	13.8	58.1
rs12449331	17	C	T	0.04	0.01	12.1	46.7
rs148898583	19	G	C	-0.14	0.01	52	235.1
rs1065853	19	T	G	-0.47	0.01	435.2	2199.6
rs6513673	20	C	T	-0.04	0.01	11.1	46.7
rs2868346	20	T	C	-0.05	0.01	12	45.1
**PRSS8**							
rs1260326	2	C	T	-0.06	0.01	26.9	110.3
rs12916	5	C	T	-0.05	0.01	15.6	61.4
6:32456105	6	G	GCTA	0.07	0.01	19.1	91.6
rs1337775	6	G	A	0.05	0.01	15.4	61.4
rs33951980	7	T	C	-0.10	0.01	33.3	162.6
rs34087092	8	A	G	0.05	0.01	14.6	57.3
rs174576	11	A	C	0.04	0.01	11.5	46.7
rs3743887	16	A	G	-0.18	0.01	65.9	273.8
rs59735493	16	A	G	0.11	0.01	72.1	348.4
rs367776888	16	A	G	0.47	0.07	11.4	48.6
rs186021206	17	A	G	0.28	0.04	12	51.9
rs55864353	17	G	T	0.05	0.01	18.1	69.4
rs57822461	19	T	C	-0.08	0.01	30.1	144.0
rs4002471	19	T	C	0.04	0.01	13.9	53.8
**PTPRZ1**							
rs1984769	1	A	G	0.08	0.01	23.5	105.1
rs9270487	6	G	A	-0.05	0.01	14.4	58.8
rs142988850	7	G	A	0.31	0.04	13.8	59.3
rs772220206	7	A	AC	-0.18	0.01	214.6	930.3
rs5894469	8	AAAACAAAC	AAAAC	0.05	0.01	18.8	69.4
rs10956755	8	G	A	-0.10	0.01	65.1	289.0

rs2519093	9	T	C	-0.06	0.01	16	71.0
rs56278466	10	G	T	0.08	0.01	43.1	177.8
rs149363012	12	T	C	0.13	0.02	14.1	62.1
rs548589580	13	TA	T	0.06	0.01	26.5	100.0
rs373909380	16	T	A	0.11	0.01	57.1	251.4
rs186021206	17	A	G	0.69	0.04	70.9	315.7
rs12451299	17	C	G	0.05	0.01	13.6	66.7
rs983817	17	T	C	0.05	0.01	12.3	55.2
rs55639531	18	G	C	0.04	0.01	11	49.0
rs601338	19	A	G	0.11	0.01	80.8	441.0

SNP, single nucleotide polymorphism; se, standard error.

*For SNPs lacking an assigned reference SNP ID number (rsID), the location notation is used, formatted as 'chromosome: position'. This format indicates the specific chromosome number followed by the precise position of the SNP on that chromosome, measured in base pairs.

†The -log(p-value) values represent the negative logarithm (to base 10) of the p-values.

Supplemental Table 3. Association of selected proteins with IHD in CARDIoGRAMplusC4D using different MR analytic methods

Exposure	Method	Log Odds Ratio (95% CI)	p-value
ANGPTL1	Weighted median	-0.07 (-0.12, -0.01)	0.02
	Weighted mode	-0.07 (-0.12, -0.01)	0.02
	MR-PRESSO	-0.07 (-0.19, 0.05)	0.19
ASGR1	Weighted median	-0.09 (-0.21, 0.03)	0.15
	Weighted mode	0.01 (-0.11, 0.14)	0.82
	MR-PRESSO	-0.17 (-0.37, 0.03)	0.07
COL6A3	Weighted median	0.22 (0.06, 0.38)	0.01
	Weighted mode	0.26 (0.08, 0.45)	4.00E-03
	MR-PRESSO	0.17 (-0.03, 0.38)	0.08
EFNA4	Weighted median	0.09 (-0.05, 0.23)	0.19
	Weighted mode	0.05 (-0.07, 0.18)	0.41
	MR-PRESSO	0.17 (0.08, 0.25)	0.01
FGFBP1	Weighted median	0.37 (0.24, 0.50)	2.86E-08
	Weighted mode	0.33 (0.22, 0.44)	5.94E-09
	MR-PRESSO	0.27 (0.03, 0.52)	0.04
PLA2G7	Weighted median	0.64 (0.48, 0.80)	2.21E-15
	Weighted mode	0.69 (0.25, 1.12)	2.00E-03
	MR-PRESSO	0.59 (0.41, 0.77)	2.08E-05
PRSS8	Weighted median	0.06 (-0.04, 0.17)	0.23
	Weighted mode	0.06 (-0.05, 0.16)	0.28
	MR-PRESSO	0.10 (0.01, 0.19)	0.04
PTPRZ1	Weighted median	0.00 (-0.08, 0.08)	1.00
	Weighted mode	0.00 (-0.07, 0.08)	0.96
	MR-PRESSO	0.01 (-0.06, 0.08)	0.71

Supplemental Table 4. Another set of genetic instruments for PLA2G7 derived from deCODE

SNP*	chr	effect allele	other allele	beta	se	-log(p-value)†	F statistics
rs11591147	1	T	G	-0.34	0.04	16.8	72.5
rs553741	1	C	G	0.05	0.01	7.6	31.1
rs599839	1	A	G	0.10	0.01	20.9	91.4
rs12029785	1	A	G	-0.06	0.01	11.8	50.1
rs12026141	1	T	C	-0.07	0.01	14.1	60.4
rs934197	2	A	G	0.07	0.01	12.6	60.1
rs62182252	2	A	G	-0.07	0.01	15.8	69.0
rs111696008	2	A	G	-0.30	0.03	7.9	111.5
rs144202860	2	A	G	-0.32	0.04	22.6	73.5
rs3863090	3	T	G	0.12	0.01	15.7	77.4
rs11720167	3	G	T	0.35	0.01	111.2	1057.3
rs148077724	3	G	A	0.31	0.03	34.3	134.4
rs111266377	3	T	G	0.24	0.03	9.5	88.1
rs147033786	3	C	A	0.37	0.04	14.0	78.1
rs9881140	3	C	T	0.12	0.02	16.0	41.0
rs6556405	5	C	T	0.06	0.01	25.3	40.9
rs4151657	6	C	T	-0.08	0.01	17.0	71.3
rs9271589	6	A	G	-0.06	0.01	17.9	36.4
rs115337345	6	G	A	-0.39	0.04	231.2	79.3
rs78696530	6	A	G	-0.19	0.03	30.4	48.0
rs139434889	6	T	C	-0.47	0.04	20.2	135.8
rs71566584	6	C	A	0.17	0.02	18.0	76.9
rs72869765	6	C	T	-0.24	0.04	9.8	44.7
rs115054514	6	T	A	-0.39	0.04	9.8	125.3
rs116824493	6	T	G	-0.46	0.04	16.5	145.9
rs12527048	6	T	C	-0.13	0.02	8.8	31.5
rs35705010	6	T	A	-0.39	0.04	18.3	94.0
rs543311582	6	T	G	-0.22	0.04	11.4	35.0
rs17145750	7	T	C	0.07	0.01	30.7	31.6
rs174576	11	A	C	-0.06	0.01	17.7	53.3
rs964184	11	C	G	-0.11	0.01	10.6	68.0
rs11057840	12	C	A	0.08	0.01	28.4	32.6
rs17248720	19	T	C	-0.15	0.01	32.9	99.0
rs4803748	19	ATTTGTAGA	ATCTGTAGA	-0.07	0.01	7.7	67.5
rs429358	19	C	T	0.25	0.01	21.5	505.2
rs144311893	19	T	C	-0.36	0.03	8.5	152.4
rs4803776	19	C	T	-0.05	0.01	7.7	39.6

SNP, single nucleotide polymorphism; eaf, effect allele frequency; se, standard error.

* For SNPs lacking an assigned reference SNP ID number (rsID), the location notation is used, formatted as 'chromosome: position'. This format indicates the specific chromosome number followed by the precise position of the SNP on that chromosome, measured in base pairs.

† The -log(p-value) values represent the negative logarithm (to base 10) of the p-values.

Supplemental Table 5. Replication of the association of genetically predicted PLA2G7 with IHD using another set of genetic instruments for PLA2G7 in CARDIoGRAMplusC4D

Method	Log Odds Ratio (95% CI)	p-value
Inverse variance weighted	0.17 (0.08, 0.27)	2.62E-04
Weighted median	0.07 (0.01, 0.13)	0.02
Weighted mode	0.05 (0.00, 0.11)	0.05
MR-PRESSO*	0.15 (0.05, 0.24)	0.01

* Outliers rs11591147, rs11720167, rs139434889, rs144311893, rs17248720, rs4151657, rs429358, and rs599839 were identified and excluded from the MR-PRESSO analysis.

Supplemental Table 6. Outliers identified in MR-PRESSO analysis

Exposure	Outcome	Data source	Outlier
ASGR1	IHD	CARDIoGRAMplusC4D	rs7528419, rs1801689, rs55714927, rs149092047
EFNA4	IHD	CARDIoGRAMplusC4D	rs148289726, rs3184504, rs4760
FGFBP1	IHD	CARDIoGRAMplusC4D	rs7528419, rs3184504, rs10402112, rs7412, rs1260326, rs6800909, rs9968276
PRSS8	IHD	CARDIoGRAMplusC4D	rs174576, rs12916
PTPRZ1	IHD	CARDIoGRAMplusC4D	rs983817, rs2519093
PLA2G7	IHD	CARDIoGRAMplusC4D	rs7528419, rs562028, rs12449331, rs148898583, rs2868346
PLA2G7	T2D	DIAGRAM	rs964184, rs17671591, rs327
		FinnGen	rs17671591, rs327
PLA2G7	eGFR	CKDGen	rs7528419, rs562028, rs2954021, rs10504255
COL6A3	T2D	DIAGRAM	rs186021206
COL6A3	CKD	CKDGen	rs1050785, rs34262842, rs112153300
EFNA4	T2D	DIAGRAM	rs3184504, rs9928936, rs10224210
EFNA4	CKD	CKDGen	rs9928936, rs11248930, rs12995875, rs10224210
EFNA4	eGFR	CKDGen	rs9928936 rs11248930, rs12995875, rs10224210

* This MR analysis was conducted using the alternative set of genetic instruments of PLA2G7.

Supplemental Table 7. Total effect of statins on ischemic heart disease

Data sources	Odds ratio (95% CI)	P value
CARDIoGRAMplusC4D	0.64 (0.51, 0.80)	7.2×10^{-5}
FinnGen	0.57 (0.43, 0.77)	2.4×10^{-4}
Meta-analysis	0.61 (0.51, 0.73)	7.5×10^{-8}

Supplemental Table 8. Associations of genetically predicted IHD and selected proteins using different analytic methods

Outcome	Method	Beta (95% CI)	p-value
COL6A3	Inverse variance weighted	0.01 (-0.04, 0.05)	0.75
	Weighted median	0.01 (-0.05, 0.06)	0.76
	Weighted mode	0.03 (-0.04, 0.10)	0.38
	MR-PRESSO	0.01 (-0.03, 0.05)	0.55
EFNA4	Inverse variance weighted	0.00 (-0.05, 0.06)	0.88
	Weighted median	0.01 (-0.05, 0.06)	0.79
	Weighted mode	0.01 (-0.06, 0.09)	0.69
	MR-PRESSO	0.00 (-0.03, 0.04)	0.82
FGFBP1	Inverse variance weighted	0.02 (-0.11, 0.14)	0.82
	Weighted median	-0.09 (-0.14, -0.03)	3.72E-03
	Weighted mode	-0.19 (-0.32, -0.05)	0.01
	MR-PRESSO	-0.06 (-0.11, -0.01)	0.01
PLA2G7	Inverse variance weighted	0.19 (0.02, 0.37)	0.03
	Weighted median	-0.02 (-0.07, 0.04)	0.58
	Weighted mode	-0.03 (-0.09, 0.03)	0.38
	MR-PRESSO	0.09 (0.02, 0.15)	0.01

Supplemental Table 9. Power calculation

Exposure	Outcomes	Data source	Effect size detected (OR for IHD, T2D and CKD; beta coefficient for eGFR)
ANGPTL1	IHD	CARDIoGRAMplusC4D	1.05
		FinnGen	1.08
ASGR1	IHD	CARDIoGRAMplusC4D	1.07
		FinnGen	1.10
COL6A3	IHD	CARDIoGRAMplusC4D	1.13
		FinnGen	1.24
EFNA4	IHD	CARDIoGRAMplusC4D	1.10
		FinnGen	1.21
FGFBP1	IHD	CARDIoGRAMplusC4D	1.06
		FinnGen	1.10
PCOLCE	IHD	CARDIoGRAMplusC4D	1.11
		FinnGen	1.16
PLA2G7	IHD	CARDIoGRAMplusC4D	1.07
		FinnGen	1.11
PRSS8	IHD	CARDIoGRAMplusC4D	1.08
		FinnGen	1.12
PTPRZ1	IHD	CARDIoGRAMplusC4D	1.07
		FinnGen	1.11
PLA2G7	T2D	DIAGRAM	1.07
	T2D	FinnGen	1.12
PLA2G7	CKD	CKDGen	1.10
PLA2G7	eGFR	CKDGen	0.03
COL6A3	T2D	DIAGRAM	1.11
		FinnGen	1.23
COL6A3	CKD	CKDGen	1.13
COL6A4	eGFR	CKDGen	0.03
EFNA4	T2D	DIAGRAM	1.09
		FinnGen	1.20
EFNA4	CKD	CKDGen	1.17
EFNA4	eGFR	CKDGen	0.04

For binary outcomes (IHD, type 2 diabetes and cancer), power calculation was based on the ratio of cases to the controls and the variance explained by the genetic proxies (r^2) (Burgess 2014). For continuous outcomes, power calculation was based on the number of participants in the study and the variance explained by the genetic proxies (Freeman, Cowling et al. 2013). The calculation of r^2 was based on 2×effect allele frequency (EAF)× (1-EAF) ×beta^2. The effect size shown in this table was in the scale of odds ratio of IHD, type 2 diabetes and cancer, and SD of the other continuous outcomes.

Supplemental Table 10. Associations of genetically predicted COL6A3 and EFNA4 with diabetes, CKD and kidney function (eGFR) using different MR analytic methods

Exposure	Outcome	Data source	Method	Log Odds ratio or beta*	se	p-value
COL6A3	T2D	DIAGRAM	IVW	-0.12	0.09	0.15
			Weighted median	-0.12	0.06	0.05
			Weighted mode	-0.08	0.09	0.36
			MR-PRESSO	-0.18	0.08	0.08
		FinnGen	IVW	-0.23	0.13	0.08
			Weighted median	-0.31	0.12	0.01
			Weighted mode	-0.39	0.17	0.02
			MR-PRESSO	-0.23	0.13	0.16
		meta-analysis	IVW	-0.16	0.07	0.03
			Weighted median	-0.19	0.09	0.04
			Weighted mode	-0.20	0.15	0.19
			MR-PRESSO	-0.19	0.07	0.00
	CKD	CKDGen	IVW	0.89	0.61	0.14
			Weighted median	0.09	0.22	0.68
			Weighted mode	0.13	0.10	0.20
			MR-PRESSO	1.90	0.32	0.10
	eGFR	CKDGen	IVW	-0.05	0.04	0.14
			Weighted median	0.00	0.01	0.81
			Weighted mode	0.00	0.00	0.41
EFNA4	T2D	DIAGRAM	IVW	0.01	0.08	0.89
			Weighted median	0.07	0.06	0.20
			Weighted mode	0.09	0.06	0.16
			MR-PRESSO	0.04	0.06	0.55
		FinnGen	IVW	0.01	0.11	0.94
			Weighted median	0.01	0.11	0.94
			Weighted mode	0.14	0.17	0.41
			MR-PRESSO	0.01	0.11	0.94
		meta-analysis	IVW	0.01	0.07	0.88
			Weighted median	0.06	0.05	0.24
			Weighted mode	0.10	0.06	0.11
			MR-PRESSO	0.03	0.05	0.54
	CKD	CKDGen	IVW	0.85	0.50	0.09
			Weighted median	0.47	0.13	0.00
			Weighted mode	0.18	0.10	0.07
			MR-PRESSO	0.53	0.15	0.18
	eGFR	CKDGen	IVW	-0.04	0.03	0.09
			Weighted median	-0.02	0.01	0.00
			Weighted mode	-0.01	0.00	0.06
			MR-PRESSO	-0.02	0.01	0.24

*log odds ratio for diabetes and CKD, and beta coefficient for eGFR were reported.

Reference:

Burgess, S. (2014). "Sample size and power calculations in Mendelian randomization with a single instrumental variable and a binary outcome." Int J Epidemiol **43**(3): 922-929.

Freeman, G., B. J. Cowling and C. M. Schooling (2013). "Power and sample size calculations for Mendelian randomization studies using one genetic instrument." Int J Epidemiol **42**(4): 1157-1163.

Mahajan, A., D. Taliun, M. Thurner, N. R. Robertson, J. M. Torres, N. W. Rayner, A. J. Payne, V. Steinthorsdottir, R. A. Scott and N. Grarup (2018). "Fine-mapping type 2 diabetes loci to single-variant resolution using high-density imputation and islet-specific epigenome maps." Nat Genet **50**(11): 1505-1513.
Nelson, C. P., A. Goel, A. S. Butterworth, S. Kanoni, T. R. Webb, E. Marouli, L. Zeng, I. Ntalla, F. Y. Lai and J. C. Hopewell (2017). "Association analyses based on false discovery rate implicate new loci for coronary artery disease." Nat Genet **49**(9): 1385-1391.
Wuttke, M., Y. Li, M. Li, K. B. Sieber, M. F. Feitosa, M. Gorski, A. Tin, L. Wang, A. Y. Chu and A. Hoppmann (2019). "A catalog of genetic loci associated with kidney function from analyses of a million individuals." Nat Genet **51**(6): 957-972.

STROBE-MR checklist of recommended items to address in reports of Mendelian randomization studies[1 2]

Item No.	Section	Checklist item	Page No.	Relevant text from manuscript
1	**TITLE and ABSTRACT**	Indicate Mendelian randomization (MR) as the study's design in the title and/or the abstract if that is a main purpose of the study	2	Abstract<br>To examine the mechanisms through proteins, we used two-step Mendelian randomization (MR) approach…
	**INTRODUCTION**			
2	**Background**	Explain the scientific background and rationale for the reported study. What is the exposure? Is a potential causal relationship between exposure and outcome plausible? Justify why MR is a helpful method to address the study question	3-4	(Introduction paragraph 1) "Ischemic heart disease (IHD) is the single leading cause of mortality and poses a heavy burden on healthcare.1 … statins can effectively lower cardiovascular mortality,5-7 with superior efficacy in reducing cardiovascular mortality than other lipid-lowering drugs, such as ezetimibe7 8 and cholestyramine.8 … In all international guidelines, there is a consensus on the use of statins as the first-line treatment in primary and secondary prevention of atherosclerotic cardiovascular disease.9 11"<br>(Introduction paragraph 2) "Despite its wide use, the specific mechanism by which statins exert cardiovascular benefits have not been fully clarified… Clarifying the mechanisms of statins is vital for understanding the pleiotropic effect of statins, and provides novel insights to new modifiable targets for drug development. Proteins are downstream factors of gene expression, play an important role in etiology of disease, including IHD,13 and are often used as drug targets for treatment. However, the specific proteins mediating the cardiovascular benefits of statins have not been examined. Mechanistic, large-scale randomized controlled trials (RCTs) would be ideal to answer this question, but they are not available yet."<br>(Introduction paragraph 3) "In this situation, Mendelian randomization (MR) studies, which can foreshadow the results of RCTs, could provide an alternative approach.14 … However, no MR study has examined the molecular

				pathways underlying the protective effects of statins. The emerging large genome-wide association studies (GWAS) of proteins, such as GWAS of 2,923 proteins in 54,219 participants in UK Biobank Pharma Proteomics Project (UKB-PPP),22 and GWAS of 4,907 proteins in 35,559 Icelanders from deCODE,23 provided a valuable opportunity to explore the mechanisms underlying the benefits of statins on IHD.”
3	**Objectives**	State specific objectives clearly, including pre-specified causal hypotheses (if any). State that MR is a method that, under specific assumptions, intends to estimate causal effects	4	(Introduction paragraph 3) “…to explore the mechanisms underlying the benefits of statins on IHD.”
	**METHODS**			
4	**Study design and data sources**	Present key elements of the study design early in the article. Consider including a table listing sources of data for all phases of the study. For each data source contributing to the analysis, describe the following:		
	a)	Setting: Describe the study design and the underlying population, if possible. Describe the setting, locations, and relevant dates, including periods of recruitment, exposure, follow-up, and data collection, when available.	4, Figure 1	(Methods, Study design) “We used two-step MR to identify proteins on the pathway from statins to IHD. First, we conducted MR analysis on the associations of genetically mimicked statins with 2,923 proteins measured in UKB-PPP, to identify the proteins affected by statins. For replication, we examined the associations of genetically mimicked statins with selected proteins using another GWAS from deCODE.23… The flow chart was shown in Figure 1.”
	b)	Participants: Give the eligibility criteria, and the sources and methods of selection of participants. Report the sample size, and whether any power or sample size calculations were carried out prior to the main analysis	4-7, Supplemental Table 1, Supplemental Table 9	(Methods, Study design) “The data sources we used were shown in Supplemental Table 1.”<br>(Methods, Associations of genetic proxies for statins with proteins) “…UK Biobank is a large, population-based cohort in UK, with around 500,000 participants aged 40 to 69 years, recruited between 2006 and 2010.26 UKB-PPP is a precompetitive consortium… in 54,219 UKB participants. The details of UKB-PPP were shown in the previous publication.22 ”<br>(Methods, Associations of genetic proxies for statins with proteins) “For replication, we used another GWAS in deCODE,23 a GWAS of

			4,907 proteins in 35,559 Icelanders, to examine the role of statins on selected protein(s).”<br><br>(Methods, The effect of selected proteins on the risk of IHD) “Then we obtained the genetic associations with IHD, using a GWAS meta-analysis from Coronary ARtery DIsease Genome wide Replication and Meta-analysis (CARDIoGRAM) plus The Coronary Artery Disease (C4D) Genetics (CARDIoGRAMplusC4D) … and the Myocardial Infarction Genetics and CARDIoGRAM Exome (42,335 cases and 78,240 controls).24 We also replicated the analysis using FinnGen (30,952 cases and 187,840 controls), and combined the MR estimates from the two data sources.”<br><br>(Methods, The effects of the identified protein(s) on type 2 diabetes, CKD and eGFR) “Genetic associations with diabetes were obtained from DIAGRAM (74,124 cases and 824,006 controls)34 and FinnGen (35,607 cases and 183,185 controls) …The GWAS meta-analysis of CKD included 41,395 cases and 439,303 controls in 23 studies; all are of European ancestry.35 The GWAS meta-analysis of eGFR included up to 567,460 participants in 42 studies.35”
	c) Describe measurement, quality control and selection of genetic variants	4-6, Supplemental Table 2, Supplemental Table 4	(Methods, Associations of genetic proxies for statins with proteins) “To ensure the genetic variants are independent, as previously,20 we selected SNPs not in linkage disequilibrium (LD) (r^2<0.05), and used the lead SNP (rs12916) as the genetic instrument.”<br><br>(Methods, The effect of selected proteins on the risk of IHD) “The genetic variants for each selected protein were obtained from the published study in Nature,22 which provided SNPs strongly associated with the proteins ($p<1.7\times10^{-11}$) and not in linkage disequilibrium (r^2<0.01).”<br><br>(Methods, Sensitivity analyses) “For replication, we used another set of genetic instruments for the selected proteins, where we selected SNPs with genome-wide significance, and not in

					linkage disequilibrium (r^2<0.001) from the GWAS of proteins in deCODE.23" (Methods, Sensitivity analyses) We also conducted reverse MR analyses for the proteins associated with IHD or having suggestive associations with IHD... To build the genetic instrument for IHD, we selected SNPs with genome-wide significance and not in linkage disequilibrium (r2<0.001) in the GWAS of IHD…
		d)	For each exposure, outcome, and other relevant variables, describe methods of assessment and diagnostic criteria for diseases	5	(Methods, Associations of genetic proxies for statins with proteins) "A total of 2,923 unique proteins across eight protein panels (cardiometabolic, cardiometabolic II, inflammation, inflammation II, neurology, neurology II, oncology and oncology II) in the antibody-based Olink Explore 3072 platform were measured, with quality control.22 One protein (GLIPR1) failed quality control and was excluded,22 so 2,922 proteins were included in the analysis."
		e)	Provide details of ethics committee approval and participant informed consent, if relevant	NA	The study used publicly available data, so ethical approval is not needed.
5	**Assumptions**		Explicitly state the three core IV assumptions for the main analysis (relevance, independence and exclusion restriction) as well assumptions for any additional or sensitivity analysis	6,11	(Methods, Sensitivity anlaysis) "For proteins with three or more genetic variants as instruments, we also used different MR methods under different assumptions from IVW, including weighted median, weighted mode and Mendelian Randomization Pleiotropy RESidual Sum and Outlier (MR-PRESSO).… and provide corrected estimates removing the outliers." (Discussion, paragraph 4) "First, MR is based on three core assumptions, i.e., the relevance, independence, and exclusion-restriction assumption.50"
6	**Statistical methods: main analysis**		Describe statistical methods and statistics used		
		a)	Describe how quantitative variables were handled in the analyses (i.e., scale, units, model)	6	(Methods, The effect of selected proteins on the risk of IHD) "Multiplicative random effects model was used when three or more genetic variants were used as instruments, and fixed

					effects model was used when less than three genetic variants were used as instruments.”
		b)	Describe how genetic variants were handled in the analyses and, if applicable, how their weights were selected	4-5	(Methods, Associations of genetic proxies for statins with proteins) “As previously,19, 20 genetic proxies for statins were genetic variants in the drug target gene, HMGCR, and also associated with lower LDL-cholesterol… as previously,20 we selected SNPs not in linkage disequilibrium (LD) (r2<0.05), and used the lead SNP (rs12916) as the genetic instrument.<br><br>In the first-step MR, we obtain Wald ratio, i.e., the genetic association with each protein divided by the association with the reduction in LDL-cholesterol. As statins are lipid-lowering drugs, the reduction in LDL-cholesterol is used as a scale to measure the effects of statins.”
		c)	Describe the MR estimator (e.g. two-stage least squares, Wald ratio) and related statistics. Detail the included covariates and, in case of two-sample MR, whether the same covariate set was used for adjustment in the two samples	6	(Methods, The effect of selected proteins on the risk of IHD) “In the second-step MR, we obtained Wald ratio estimates for each SNP, using genetic associations with IHD divided by genetic associations with each protein, and meta-analysed these estimates using inverse variance weighting (IVW). Multiplicative random effects model was used when three or more genetic variants were used as instruments, and fixed effects model was used when less than three genetic variants were used as instruments. To control for multiple testing, we also selected proteins associated with IHD with FDR<0.05.”
		d)	Explain how missing data were addressed	N/A	
		e)	If applicable, indicate how multiple testing was addressed	5	(Methods, Associations of genetic proxies for statins with proteins) “As we did multiple testing, to control the ratio of false positive results to true positive results within a certain range, we selected proteins with false discovery rate (FDR)<0.05.”
7	**Assessment of assumptions**		Describe any methods or prior knowledge used to assess the assumptions or justify their validity	4-5, 7	(Methods, Study design) “To test whether the identified target(s) have pleiotropic effects on common comorbidities of IHD, including diabetes and chronic kidney disease (CKD), we also examined the effects of the target(s) on

				diabetes, CKD and kidney function (indicated by eGFR)."<br>(Methods, Associations of genetic proxies for statins with proteins) "To ensure the genetic variants are independent, as previously,20 we selected SNPs not in linkage disequilibrium (LD) (r^2<0.05), and used the lead SNP (rs12916) as the genetic instrument."<br>(Methods, The effect of selected proteins on the risk of IHD) "The genetic variants for each selected protein were obtained from the published study in Nature,22 which provided SNPs strongly associated with the proteins ($p<1.7\times10^{-11}$) and not in linkage disequilibrium (r2<0.01)."<br>(Methods, The effects of the identified protein(s) on type 2 diabetes, CKD and eGFR) "For the identified mediating proteins, to examine their pleiotropic effects, we also examined their associations with diabetes, CKD and eGFR."
8	**Sensitivity analyses and additional analyses**	Describe any sensitivity analyses or additional analyses performed (e.g. comparison of effect estimates from different approaches, independent replication, bias analytic techniques, validation of instruments, simulations)	6-7	(Methods, Sensitivity analyses) "For replication, we used another set of genetic instruments for the selected proteins…from the GWAS of proteins in deCODE.23 In sensitivity analysis, to visualize the potential outliers, we used leave-one-out analysis and scatter plots… we also used different MR methods under different assumptions from IVW, including weighted median, weighted mode and Mendelian Randomization Pleiotropy RESidual Sum and Outlier (MR-PRESSO)…provide corrected estimates removing the outliers.<br>We also conducted reverse MR analyses for the proteins associated with IHD or having suggestive associations with IHD, to examine whether genetically predicted IHD was associated with the proteins… In the reverse MR, similarly we used IVW, weighted median, weighted mode and MR-PRESSO. We also used Steiger test which can infer the causal direction, by calculating and comparing the variance explained by the genetic instrument in

					exposure (here IHD) and in outcome (here the proteins).30<br>To exclude the possible confounding by genotype, we conducted colocalization analysis for the effect of statins on the selected protein… A high PPH3 suggests confounding.32"<br>(Methods, The effects of the identified protein(s) on type 2 diabetes, CKD and eGFR) "Similar to prior analyses, we used IVW in the main analyses, supplemented by sensitivity analyses using weighted median, weighted mode and MR-PRESSO."
9	**Software and pre-registration**				
		a)	Name statistical software and package(s), including version and settings used	8	(Methods) "All statistical analyses were conducted using the "TwoSampleMR",36 "MendelianRandomization",37 "MRPRESSO"38 and "coloc"31 packages in R (version 4.0.1, R Foundation for Statistical Computing, Vienna, Austria)."
		b)	State whether the study protocol and details were pre-registered (as well as when and where)	N/A	
	**RESULTS**				
10	**Descriptive data**				
		a)	Report the numbers of individuals at each stage of included studies and reasons for exclusion. Consider use of a flow diagram	Figure 1, Supplemental Table 1	Figure 1. Flow chart of the two-step MR study design to identify proteins on the pathway from statins to IHD<br>Supplemental Table 1. Overview of genome-wide association studies incorporated in this research
		b)	Report summary statistics for phenotypic exposure(s), outcome(s), and other relevant variables (e.g. means, SDs, proportions)	N/A	
		c)	If the data sources include meta-analyses of previous studies, provide the assessments of heterogeneity across these studies	N/A	
		d)	For two-sample MR:	11	(Discussion, paragraph 4) "Third, it is possible that a small proportion of samples overlap in the GWAS of proteins and GWAS of outcomes

		i. Provide justification of the similarity of the genetic variant-exposure associations between the exposure and outcome samples<br>ii. Provide information on the number of individuals who overlap between the exposure and outcome studies		(such as GWAS from CARDIoGRAMplusC4D, DIAGRAM, CKD and eGFR), which both included UK Biobank. The sample overlapping may bias the estimates in the MR analyses on the role of proteins in the outcomes,51 however, a simulation study has shown that it is not a major concern if MR is conducted in large cohorts, such as UK Biobank.52 There is no overlapping in the GWAS of exposure (i.e., the GWAS used for building genetic instrument for statins, from GLGC) and GWAS of the outcomes.”
11	**Main results**			
		a) Report the associations between genetic variant and exposure, and between genetic variant and outcome, preferably on an interpretable scale	8, Supplemental Table 2	(Results, The effect of selected proteins on the risk of IHD) “Then we examined the effect of the 9 proteins identified in UKB-PPP on IHD. All the SNPs for these selected proteins have F-statistics above 10 (genetic instruments shown in Supplemental Table 2).”
		b) Report MR estimates of the relationship between exposure and outcome, and the measures of uncertainty from the MR analysis, on an interpretable scale, such as odds ratio or relative risk per SD difference	8-9, Figure 3-4, Supplemental Table 10	(Results, The effect of selected proteins on the risk of IHD) “Of these 9 proteins, after multiple testing correction… PLA2G7, remained to be related to IHD … The positive association with IHD was replicated in FinnGen and in the meta-analysis of both GWAS (Figure 3). Three proteins (FGFBP1, COL6A3 and EFNA4) had suggestive associations with IHD (Figure 3)… PLA2G7 and FGFBP1 were related to higher risk of IHD and decreased by statins, consistent with statins lowering the risk of IHD. COL6A3 and EFNA4 were related to higher risk of IHD, and were increased by statins...”<br>(Results, The effects of the identified protein(s) on type 2 diabetes, CKD and eGFR) “When assessing the role of PLA2G7 in diabetes, CKD and kidney function (eGFR), we found genetically predicted PLA2G7 was not related to diabetes, CKD and eGFR using IVW, and these findings were robust across different analytic methods (Figure 4).”<br>(Results, The effects of the identified protein(s) on type 2 diabetes, CKD and eGFR) “Genetically predicted COL6A3 was associated with lower risk of diabetes using IVW and

					weighted median, EFNA4 was related to higher risk of CKD and lower eGFR using weighted median, but the findings were not shown in other methods (Supplemental Table 10)."
		c)	If relevant, consider translating estimates of relative risk into absolute risk for a meaningful time period	N/A	
		d)	Consider plots to visualize results (e.g. forest plot, scatterplot of associations between genetic variants and outcome versus between genetic variants and exposure)	8-9, Figure 3-4	(Results, The effect of selected proteins on the risk of IHD) "Three proteins (FGFBP1, COL6A3 and EFNA4) had suggestive associations with IHD (Figure 3)…"<br>(Results, The effects of the identified protein(s) on type 2 diabetes, CKD and eGFR) "When assessing the role of PLA2G7 in diabetes…findings were robust across different analytic methods (Figure 4)."
12	**Assessment of assumptions**				
		a)	Report the assessment of the validity of the assumptions	8-9	(Results, The effect of selected proteins on the risk of IHD) "The associations were robust to different analytic methods …was replicated in FinnGen and in the meta-analysis of both GWAS (Figure 3). In the replication using another set of genetic instruments derived from deCODE … was also replicated … MR-PRESSO estimates removing the outliers showed consistent estimates as in the main analyses (Supplemental Table 3)."<br>(Results, The effects of the identified protein(s) on type 2 diabetes, CKD and eGFR) "When assessing the role of PLA2G7 in diabetes, CKD and kidney function (eGFR), we found genetically predicted PLA2G7 was not related to diabetes, CKD and eGFR using IVW, and these findings were robust across different analytic methods (Figure 4)."
		b)	Report any additional statistics (e.g., assessments of heterogeneity across genetic variants, such as I^2, Q statistic or E-value)	N/A	
13	**Sensitivity analyses and additional analyses**				

	a)	Report any sensitivity analyses to assess the robustness of the main results to violations of the assumptions	8-9, Supplemental Table 3, Figure 3-4	(Results, The effect of selected proteins on the risk of IHD) “The associations were robust to different analytic methods (Supplemental Table 3). The positive association with IHD was replicated in FinnGen and in the meta-analysis of both GWAS (Figure 3).”<br><br>(Results, The effects of the identified protein(s) on type 2 diabetes, CKD and eGFR) “… and these findings were robust across different analytic methods (Figure 4).”
	b)	Report results from other sensitivity analyses or additional analyses	8-9, Table 2, Supplemental Table 4-5	(Results, Associations of genetic proxies for statins with proteins) “In the replication analysis… we replicated the associations of statins with 4 proteins, including PLA2G7, FGFBP1, PRSS8 and PCOLCE (Table 2).”<br><br>(Results, The effect of selected proteins on the risk of IHD) “In the replication using another set of genetic instruments derived from deCODE (Supplemental Table 4), the positive association of PLA2G7 with IHD was also replicated (Supplemental Table 5).<br><br>In colocalization analysis, the small PPH3 (0.01) did not suggest the effect of statins on PLA2G7 is due to confounding by genotype. The proportion mediated by PLA2G7 was 77%, calculated based on the indirect effect via PLA2G7 (log odds ratio (OR) -0.38) and total effect (log OR -0.49, shown in Supplemental Table 7).<br><br>Using a different dataset, i.e., for the effect of statins on PLA2G7, we used another dataset of PLA2G7 in deCODE, which does not overlap with UK Biobank, and for the effect of PLA2G7 on IHD, we used another dataset of IHD in FinnGen, which does not overlap with CARDIoGRAMplusC4D, we still found statins lowered PLA2G7 (Table 2), and PLA2G7 increased the risk of IHD (Figure 3), the indirect effect via PLA2G7 is log OR -0.30, and the mediation proportion is 60%.”
	c)	Report any assessment of direction of causal relationship (e.g., bidirectional MR)	9	(Results, The effect of selected proteins on the risk of IHD) “In the reverse MR analysis, based on 49 SNPs for IHD, genetically predicted IHD was not related to the four proteins associated

					with IHD or had suggestive associations with IHD using IVW after multiple testing correction (Supplemental Table 8). Genetically predicted IHD was related to lower FGFBP1 and higher PLA2G7 in sensitivity analyses (Supplemental Table 8), but the Steiger test did not support the direction from IHD to proteins.”
		d)	When relevant, report and compare with estimates from non-MR analyses	N/A	
		e)	Consider additional plots to visualize results (e.g., leave-one-out analyses)	8-9, Supplemental Figures 1-21	(Results, The effect of selected proteins on the risk of IHD) “… scatter plots (Supplemental Figures 1-9) and leave-one-out plots (Supplemental Figures 10-18) …”<br><br>(Results, The effects of the identified protein(s) on type 2 diabetes, CKD and eGFR) “Scatter plots and leave-one-out plots did not indicate outliers (Supplemental Figures 19-21).”
	**DISCUSSION**				
14	**Key results**		Summarize key results with reference to study objectives	9-10	(Discussion, paragraph 1) “Our findings added to the limited evidence on the biological mechanism underlying statins’ cardiovascular benefits, by showing that PLA2G7 possibly mediated the effect of statins on IHD. Using proteomics in the MR analysis, we found that statins lowered PLA2G7, and genetically predicted PLA2G7 was related to higher risk of IHD. The associations were replicated using another GWAS of proteomics, and using a different set of genetic instrument for PLA2G7.”<br><br>(Discussion, paragraph 3) “In addition to PLA2G7, we also identified several proteins affected by statins, and three (FGFBP1, PRSS8 and PCOLCE) were replicated using another GWAS of proteins.”
15	**Limitations**		Discuss limitations of the study, taking into account the validity of the IV assumptions, other sources of potential bias, and imprecision. Discuss both direction and magnitude of any potential bias and any efforts to address them	11-12	(Discussion, paragraph 4) “First, MR is based on three core assumptions, … To satisfy these assumptions, we used genetic variants strongly associated with these proteins. Considering the potential pleiotropy, we not only used multiple analytic methods robust to pleiotropy but also conducted replication using different datasets… The findings robust to different analytic methods and replication analyses are less like

to be due to pleiotropy. Second, population stratification might affect MR estimates. However, the GWAS data used in this study were derived from people largely of European ancestry. Meanwhile, as the study was based on people of European ancestry, the findings may not be generalizable to other ancestries. Third, it is possible that a small proportion of samples overlap in the GWAS of proteins and GWAS of outcomes … both included UK Biobank. The sample overlapping may bias the estimates in the MR analyses on the role of proteins in the outcomes,51 however, a simulation study has shown that it is not a major concern if MR is conducted in large cohorts, such as UK Biobank.52 There is no overlapping in the GWAS of exposure … and GWAS of the outcomes. Fourth, we conducted the analyses on all the proteins available in the proteomics panel, however, we did not identify apolipoprotein B (ApoB) to be affected by statins using the proteomics data in the UK Biobank (n=~35,000). Despite, when using a much larger dataset, the UK Biobank metabolomics dataset (n=~388,022), as expected, we found genetically mimicked statins lowered ApoB (beta -0.75, 95% CI -0.80 to -0.69, p value=2.8×10-146), which added validity to the genetic instrument for statins. As such, the lack of statistically significant association of the genetic instrument for statins with ApoB in the proteomics dataset may not be due to pleiotropy, but be due to the relatively small sample size of the proteomics dataset in UK Biobank. Fifth, we only identified one protein, PLA2G7, as a potential mediator after multiple testing correction, so we cannot use other related proteins to validate the result. … PLA2G7 is an enzyme involved in the metabolism of lipoprotein… The consistency from sensitivity analysis and replication analysis provided confidence to the validity of the finding. Sixth, the proportion of mediation via PLA2G7 varied when using different datasets in the analysis, which needs to be interpreted with caution. However, the mediation via PLA2G7 was replicated using studies with no overlapping. Finally, the selection of proteins

				was based on the proteins assayed in the UK Biobank. We cannot exclude the possibility that other proteins not assayed were involved in the mechanisms underlying statins' cardiovascular effects."
16	**Interpretation**			
		a) Meaning: Give a cautious overall interpretation of results in the context of their limitations and in comparison with other studies	10-11	(Discussion, paragraph 2) "Our finding of statins lowering PLA2G7 were consistent with a previous clinical trial showing PLA2G7 decreased after taking statins.39 … which used genetic instruments different from our study. There is also genetic evidence suggests that Lp-PLA2-lowering alleles were not related to the risk of IHD,41 however, this study was only conducted for each allele, rather than an MR study taking advantage of all genetic variants for PLA2G7. In addition, two large RCTs of darapladib, an Lp-PLA2 inhibitor, showed that in patients with coronary heart disease, darapladib did not reduce the risk of major coronary events.42 43 It is possible that darapladib had unidentified off-target effects which may account for the absence of efficacy in the trials.42 It is also possible that the clinical trials had a relatively short follow-up (mean follow-up time no more than 3.5 years in the two trials42 43), whilst MR examines the lifelong effect of PLA2G7."<br><br>(Discussion, paragraph 3) "The increase in PCOLCE in our study was corroborated by the clinical trial showing PCOLCE was increased by taking statins.39… involved in the pathway underlying statins' cardiovascular benefits."<br><br>(Discussion, paragraph 4) "Our study is the first study to use proteomics to examine the underlying pathways of statins' benefits on IHD… in the absence of large mechanistic RCTs."
		b) Mechanism: Discuss underlying biological mechanisms that could drive a potential causal relationship between the investigated exposure and the outcome, and whether the gene-environment equivalence assumption is reasonable. Use causal language carefully, clarifying that IV estimates may provide causal effects only under certain assumptions	10-11	(Discussion, paragraph 2) "PLA2G7 hydrolyzes and inactivates platelet-activating factor (PAF), a potent pro-inflammatory signaling lipid, and hydrolyzes oxidatively truncated phospholipids carrying an aldehyde group at omega position, preventing their accumulation in LDL

				particles.44 … On the other hand, it suggests that drugs targeting PLA2G7 may lower the risk of IHD, without increasing the risk of diabetes, with relevance to new drug development.”<br><br>(Discussion, paragraph 3) “Although there is no direct evidence on how statins affect FGFBP1, some studies suggest that statins may affect the expression of fibroblast growth factors (FGFs) and their binding proteins.47 48… the mechanism by which statins affected FGFBP1.”
	c)	Clinical relevance: Discuss whether the results have clinical or public policy relevance, and to what extent they inform effect sizes of possible interventions	10,12	(Discussion, paragraph 3) “Although they may not be the mediators for the cardiovascular benefit of statins, the identification of these targets may improve the understanding of statins’ effects, with relevance to the exploration of drug off-target effects.”<br><br>(Discussion, paragraph 5) “From the perspective of public health and clinical perspective, our study suggests that PLA2G7 possibly mediated the role of statins in IHD. As such, medications or dietary factors affecting PLA2G7 are expected to exert cardiovascular benefits. We also identified several proteins affected by statins, including four proteins replicated in a different GWAS. FGFBP1 may also be relevant to statins’ biological pathways, but the effect of FGFBP1 on IHD remains to be replicated in large GWAS of IHD.”
17	**Generalizability**	Discuss the generalizability of the study results (a) to other populations, (b) across other exposure periods/timings, and (c) across other levels of exposure	11	(Discussion, paragraph 4) “Meanwhile, as the study was based on people of European ancestry, the findings may not be generalizable to other ancestries.”
	**OTHER INFORMATION**			
18	**Funding**	Describe sources of funding and the role of funders in the present study and, if applicable, sources of funding for the databases and original study or studies on which the present study is based	13	Acknowledgements: The study received no funding.
19	**Data and data sharing**	Provide the data used to perform all analyses or report where and how the data can be accessed, and reference these sources in the article. Provide the statistical code needed to reproduce the results in the article, or report whether the code is publicly accessible and if so, where	13	Data and data sharing: Data described in the manuscript are publicly available. The data sources/references were shown in Supplemental Table 1.

20	**Conflicts of Interest**	All authors should declare all potential conflicts of interest	13	Conflicts of interest: Both authors had no conflict of interest.

This checklist is copyrighted by the Equator Network under the Creative Commons Attribution 3.0 Unported (CC BY 3.0) license.

1. Skrivankova VW, Richmond RC, Woolf BAR, Yarmolinsky J, Davies NM, Swanson SA, et al. Strengthening the Reporting of Observational Studies in Epidemiology using Mendelian Randomization (STROBE-MR) Statement. JAMA. 2021;under review.

2. Skrivankova VW, Richmond RC, Woolf BAR, Davies NM, Swanson SA, VanderWeele TJ, et al. Strengthening the Reporting of Observational Studies in Epidemiology using Mendelian Randomisation (STROBE-MR): Explanation and Elaboration. BMJ. 2021;375:n2233.
